# Stem cell activation in organ culture reveals novel transcriptional programs underlying metabolic, fibrotic, vascular, and immune dysregulation in uterine leiomyomas

**DOI:** 10.3389/fcell.2026.1804196

**Published:** 2026-04-22

**Authors:** Paula Vázquez, Ana Salas, Silvia Beltrán-Flores, Francisco Montes de Oca, Araceli Delgado, Teresa A. Almeida

**Affiliations:** 1 Molecular Bases of Neoplasms Group (MOLNEO), Department of Biochemistry, Microbiology, Cell Biology and Genetics, Universidad de La Laguna (ULL), La Laguna, Tenerife, Spain; 2 Institute of Tropical Diseases and Public Health of the Canary Islands (IUETSPC), Tenerife, Spain; 3 Canary Islands Health Research Institute Foundation (FIICS), University Hospital of Gran Canaria Dr. Negrín, Las Palmas, Spain; 4 Canary Islands Health Research Institute (IISC), Tenerife, Spain; 5 Hospital Quirónsalud Tenerife, Santa Cruz de Tenerife, Spain; 6 Department of Chemical Engineering and Pharmaceutical Technology, Universidad de La Laguna, La Laguna, Tenerife, Spain; 7 Institute of Biomedical Technologies (ITB), Universidad de La Laguna, La Laguna, Spain

**Keywords:** extracellular matrix remodeling, fibroids, fibrosis, organ culture, pathway enrichment analyses, somatic stem cell, transcriptional programs, uterine leiomyomas

## Abstract

**Background and Objective:**

Uterine leiomyomas may arise from somatic stem or progenitor cells, leading to abnormal activation, proliferation, and clonal expansion. In organ cultures of myometrium and leiomyoma, differentiated cells decline after 7 days, whereas resident stem cells may persist within their niches and subsequently become activated, proliferate, and repopulate tissue slices. This study investigated gene expression programs that regulate the proliferation and differentiation of myometrial and *MED12*-mutant leiomyoma stem cells during long-term organ culture.

**Results:**

Comparison of normal and tumor tissues at baseline and after culture revealed several fibroid transcriptional signatures that were preserved during prolonged *ex vivo* culture. The *MED12* mutation persisted in the repopulated fibroid slices, supporting the hypothesis that fibroids originate from stem or progenitor cells harboring *MED12* mutation. Both tissues activated hypoxia and stemness-associated programs, including robust induction of *HMGA1*, *HMGA2*, and *PLAG1*. Myometrium induced KITLG/KIT expression, a limited number of CD49b (ITGA2)-stem-positive and Ki67-positive proliferating cells, indicating restrained proliferation, likely mediated by upregulation of *ITGA2-AS1*. Additionally, myometrial slice cultures were enriched for immune and endothelial/vascular programs, including several SOX family members. In contrast, leiomyoma cultures exhibited widespread CD24/CD73 expression, focal CD49b clusters, high Ki67 positivity, metabolic reprogramming toward complex carbohydrate degradation, SLC-mediated transport, and a low-PLIN2/high-ACLY signature. Uterine leiomyoma cultures repressed genes involved in vascular homeostasis (e.g., *PLPP3*) and preferentially activated pathways related to smooth muscle excitability and vesicle secretion. Extracellular matrix (ECM) remodeling was strongly pro-fibrotic in leiomyomas, with significant upregulation of several TGFB-regulated and related genes, a disrupted balance of KLF regulators, including loss of the anti-fibrotic repressor *KLF10* and induction of the pro-fibrotic *KLF5* factor, and broad upregulation of integrins. Differential expression of multiple HOX genes further distinguished ECM regulation between tissues. From niche survival to pro-fibrotic expansion, the study delineates checkpoints primed for intervention, highlighting potential therapeutic opportunities targeting profibrotic signaling, metabolic dependencies, and integrin-mediated ECM interactions.

**Conclusion:**

Long-term organ culture recapitulates key molecular features of fibroids and reveals tissue-specific mechanisms governing stem cell activation and differentiation. These findings identify potential therapeutic opportunities and establish long-term organ culture as a robust, physiologically relevant platform for investigating normal and tumor biology.

## Graphical Abstract

**Figure:**
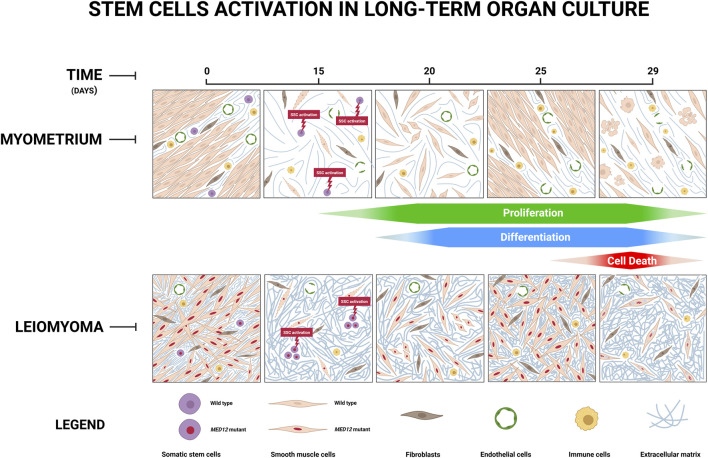
Schematic representation of cellular dynamics in long-term organ cultures of myometrium (top) and leiomyoma (bottom) over 29 days. After 7 days of culture, most differentiated cells undergo cell death, whereas somatic stem cells (SSCs) remain within their native niches. Subsequent SSC activation drives tissue repopulation through proliferation and differentiation, enabling comparative analysis of the genetic programs underlying normal versus *MED12*-mutant SSC behavior. In leiomyomas, SSC activation and expansion are associated with transcriptional programs that promote immune evasion, aberrant vascularization, and excessive extracellular matrix accumulation. At late stages, proliferative activity declines and cell death predominates. Created in BioRender. Vázquez, P. (2026) https://BioRender.com/2chunaq.

## 1 Introduction

Uterine leiomyomas (UL), also called myomas or fibroids, are benign tumors of the female genital tract, affecting approximately 70% of Caucasian and 80% of Black African women by age 50 (Stewart, 2015). Roughly 30%–50% of fibroids cause symptoms that significantly impact quality of life, with heavy or prolonged menstrual bleeding being the most common and the primary indication for surgery. Increasing evidence indicates that fibroids negatively affect fertility and reproductive outcomes, including early pregnancy loss, preterm labor, placental abnormalities, intrauterine growth restriction, and increased rates of caesarean section and postpartum hemorrhage. These adverse outcomes are thought to result from multiple mechanisms by which leiomyomas impair fertility, including uterine cavity distortion, altered uterine blood flow and contractility, hormonal and molecular changes, and reduced endometrial receptivity (Don et al., 2023; Donnez et al., 2024). Treatment typically involves myomectomy for women wishing to preserve fertility, or hysterectomy for those who have completed childbearing. Although minimally invasive alternatives exist, they are not widely adopted, and surgery remains the standard approach, contributing to substantial healthcare costs worldwide (Stewart, 2015; Cardozo et al., 2012; Soliman et al., 2015).

Although the pathogenesis of uterine leiomyomas remains under investigation, molecular data suggest distinct subgroups. Among them, UL harboring mutations in the mediator complex subunit 12 gene, *MED12*, and UL with rearrangements in the high mobility group AT-hook 2 gene, *HMGA2*, leading to increased *HMGA2* mRNA expression, account for approximately 90% of UL (Mehine et al., 2013; Mehine et al., 2016). Mutations in *MED12* occur in approximately 70% of UL, making it the most frequent subgroup, and in 99% of these tumors, the mutation occurs in exon 2 (Mäkinen et al., 2011).

Somatic stem cells (SSCs) are essential in the female reproductive tract, where they confer remarkable plasticity and exceptional regenerative potential for uterine pregnancy-induced expansion (Ono et al., 2014; Santamaria et al., 2018). A range of surface markers has been characterized in human stem/progenitor cells of the myometrium (MM), including CRIP1, Stro-1/CD44, CD34, and CD49f (Mas et al., 2015a; Ono et al., 2015; Paul et al., 2023). In UL, the most recent hypothesis regarding origin suggests that a genetic alteration, such as the *MED12* mutation, may transform a myometrial stem cell into a fibroid stem cell, initiating uncontrolled proliferation and fibrotic transformation (Bulun, 2013; Commandeur et al., 2015; Khadka et al., 2025). Accordingly, specific cell populations isolated from UL tissue that express the surface marker CD49b exhibited high levels of the stem cell transcription factors *OCT4*, *KLF4*, and *NANOG*, demonstrated the capacity to form colonies *in vitro* and regenerate tumors *in vivo*, strongly indicating that CD49b^+^ cells possess stem cell-like properties (Yin et al., 2015). In addition, UL smooth muscle cells exhibited higher expression of the CD24 surface marker (CD24^hi^) compared to normal myometrium (Drosch et al., 2014). These CD24^hi^ cells exhibit characteristics of progenitor cells, including reduced alpha-smooth muscle actin expression and elevated CD73 levels. Notably, *MED12* inhibition (mimicking *MED12* mutation) in myometrial SSCs led to the upregulation of several extracellular matrix (ECM) components, supporting that *MED12*-mutated SSCs favor fibrotic remodeling and tumor growth (Khadka et al., 2025).

Increasing evidence indicates that low-oxygen levels stimulate tissue-specific stem cell survival and growth (Ono et al., 2007; Mohyeldin et al., 2010). Notably, while myometrial SSCs demonstrated no *in vitro* proliferation in a normoxic (20% O2) environment, they exhibited robust growth under 2% oxygen tension, successfully differentiating into smooth muscle cells (SMCs). We previously established organ cultures of UL and MM, in which cells in tissue slices maintained their structural integrity and functional activity for 7 days (Salas et al., 2020). Subsequent cell death led to a dramatic reduction in the number of cells within the tissue sections. Remarkably, after 15–20 days in culture, or long-term culture (LT-culture), new cells, predominantly SMCs, emerged and repopulated the tissue slice, driven by SSC activation and subsequent differentiation (Salas et al., 2022).

The molecular mechanisms regulating activation, proliferation, and differentiation of stem cells in both myometrial and fibroid tissues remain poorly characterized. To address this knowledge gap, we compared paired myometrial and fibroid tissues from slow-proliferating leiomyomas at the time of surgery and following LT-culture. Analysis of differentially expressed genes (DEGs) across tissue types and culture conditions identified fibroid-associated transcriptional signatures that persisted under LT-culture, including pathways previously reported as dysregulated in fibroids. Furthermore, the spontaneous activation of SSCs in LT cultures enabled the identification of fibroid-specific genes involved in metabolic reprogramming, ECM remodeling and fibrosis, immune and vascular suppression, and an excitable-secretory phenotype. These findings establish UL organ culture as a platform for investigating fibroid biology and for uncovering novel molecular pathways and potential therapeutic targets relevant to *MED12*-mutated fibroids.

## 2 Materials and methods

### 2.1 Ethics approval and consent to participate

This study was approved by the Institutional Review Board of the Committee for Drug Research Ethics of the Complejo Hospitalario Universitario de Canarias (CHUC_2022_90, BioNanoGene 2022). Informed consent was obtained from the patients before FMO collected any samples. All experiments involving human tissues were performed in accordance with the principles outlined in the Declaration of Helsinki.

### 2.2 Collection of tissue samples

Four premenopausal Caucasian female patients aged 44–51 years, admitted to Hospital Quironsalud Tenerife, were enrolled in this study between January and February 2023. All patients underwent hysterectomy for irregular and heavy menstrual bleeding or bulk symptoms, and none had received hormonal treatment for at least 3 months prior to surgery. Tumors analyzed ranged in size from 5 to 8 cm, and for each sample, its paired myometrium was also collected. Histopathological analysis using standard hematoxylin and eosin (H&E) staining performed by a pathologist revealed benign tumors with no signs of malignancy, nuclear atypia, mitotic figures, or necrosis. After surgery, samples were immediately submerged in sterile Hank’s balanced salt solution (HBSS) supplemented with 0.25 μg/mL amphotericin B, 100 U/mL penicillin, and 100 μg/mL streptomycin (Sigma-Aldrich Co., United States of America). Tissue pieces were then transported to the laboratory and handled under sterile conditions. The samples were processed within 2 h after surgery.

### 2.3 Tissue sectioning

UL and MM pieces were removed from the tubes and placed in Petri dishes containing sterile, cold HBSS supplemented with antibiotics and antifungal agents. Then, a section approximately 1 cm thick and 2–3 cm^2^ was dissected and gently held between the forceps. Next, the tumor piece was cut in half using a double-edged carbon-steel blade (Ted Pella Inc., United States) to obtain two thinner sections. This procedure was repeated sequentially for each section until further halving was no longer feasible, typically resulting in tissue slices approximately 500 μm thick. Then, the slice was placed on a glass plate with a 5 × 5 mm square mark to trim and obtain pieces of similar size. The tumor slices were kept well-soaked in cold HBSS during the procedure until they were cultured.

### 2.4 Slice tissue culture

UL and MM tissue slices were cultivated onto polystyrene CELLSCAFLD® 3D (JET BIOFIL, China) and placed in a six-well culture plate. Four tissue slices were placed onto each scaffold, and 1 mL of Dulbecco’s modified Eagle’s medium (DMEM, Biowest, France) supplemented with 10% fetal bovine serum (FBS, Lonza, Spain), 100 U/mL penicillin, 100 μg/mL streptomycin, and 2 mM L-Glutamine (Sigma-Aldrich, United States) was added to each well. A medium drop was added to the tissue slice to maintain the explant’s humidity. Tissue culture plates were maintained at 37 °C in a 5% CO2 humidified incubator on an orbital shaker (60 rpm). The medium was changed every day.

### 2.5 Tissue histology characterization and cell quantification

To assess the morphological integrity of the tissue slices, two replicates of each tumor at days 0, 7, 15, 20, 25, and 29 were stained with H&E. The tissue slices were painted with green Ink for biopsies (Green Ink, VWR Q-Path Chemicals, United States) on the air-contact side before fixation to evaluate changes between the two sides of the slice. Tissues were fixed in 10% buffered formalin, then embedded in paraffin in horizontal and vertical orientations to allow for different sectioning planes, and cut in 4-μm-thick sections. The sections were deparaffinized, hydrated, and H&E-stained as described before (Salas et al., 2020).

To quantify the number of cells at each time point in culture, 3-4 random areas per sample from H&E-stained sections were captured at ×400 magnification (area = 0.0949 mm^2^) under bright field illumination using a fluorescence microscope (Leica DM4000B, Leica Microsystems, Germany), and digital images were processed in Fiji (ImageJ) (version 1.54g). First, the Hematoxylin channel (Colour_1) was separated after using the H&E vector in Color Deconvolution. Then, images were converted to 8-bit grayscale and binarized using the Threshold function. The Watershed function was applied to separate adjacent nuclei. Finally, nuclei were counted using the Analyze Particles function, applying an approximate particle size range of 50 pixels^2^ to infinity and a circularity filter of 0.2–1.0 to exclude small debris and artifacts. Automated counts were visually inspected, and ambiguous nuclei were manually verified.

### 2.6 RNA isolation

Tissue samples were placed into lysing matrix tubes D containing 500 µL of Tri-reagent (Zymo Research) and homogenized twice for 30 s at 6 m/s using a FastPrep-24TM instrument (MP Biomedicals, Illkirch, France). The sample tubes were placed on ice for 5 min between pulses. Then, chloroform was added to the lysate, the mixture was centrifuged, and the RNA from the top aqueous phase was isolated and purified using the Direct-zol RNA Microprep kit according to the manufacturer’s protocol (Zymo Research). Residual genomic DNA was removed by incubating the RNA samples with RNase-free DNase I and RNasin (Promega Corp., Madison, WI, United States) according to the manufacturer’s instructions. The effectiveness of the DNase treatment was assessed in samples with no reverse transcriptase added (RT-negative).

RNA was quantified by absorbance using a NanoDrop ND-1000 spectrophotometer (ThermoFisher Scientific, Waltham, MA, United States). Total RNA quality was assessed using an Agilent Bioanalyzer 2,100. RNA integrity number (RIN) ranged from 3.5 to 7.5.

### 2.7 *MED12* mutation detection

Two tissue slices were analysed to confirm driver genetic alterations during the culture period, one at collection time (T0) and the other after LT-culture. Amplification of *MED12* cDNA was performed with primers located in exon 1 and exon 2, covering the hot spot region where 99% of mutations have been described. PCR products were cleaned up using ExoSAP-IT (GE Healthcare Biosciences, United States) according to the manufacturer’s instructions. Sequencing reactions were performed for both strands at the Genomic Service of the University of La Laguna (SEGAI). For each sample, forward and reverse electropherograms were manually checked using Chromas v2.6.6 (Technelysium Pty Ltd, Australia).

### 2.8 *HMGA2* mRNA detection

To detect fibroids with *HMGA2* overexpression, we performed PCR using primers previously designed (Salas et al., 2020). PCR mixes contained 0.25 pmol of each primer, 1 unit of TEMPase Hot Start DNA Polymerase (VWR), 150 µM dNTPs, and 4 µL of cDNA (1/12 dilution) in a final volume of 20 µL. The cycling conditions were 95 °C for 15min, followed by 40 cycles of 95 °C for 15s, 66 °C for 20s, and 72 °C for 30s. For each experiment, a non-sample reaction and a positive control were included. The PCR products were separated by agarose gel electrophoresis, and amplicon sizes were verified by comparison with a 100-bp DNA ladder.

### 2.9 3′mRNA-Seq library preparation and sequencing

Fibroid tissue contains abundant and variable ECM, requiring intensive mechanical homogenization that can compromise RNA quality. In our sample set, RIN ranged from 3.5 to 7.5. Consequently, libraries were prepared using a 3′mRNA-Seq approach with the Lexogen QuantSeq 3′mRNA-Seq Library Prep Kit FWD. This method sequences only the region adjacent to the poly(A) tail and is commonly used in gene-expression profiling studies with limited RNA integrity, as it does not require full transcript coverage. Supporting this approach, previous studies have demonstrated that 3′mRNA-Seq methods provide reproducible gene-level quantification from degraded RNA samples, including FFPE material (Jang et al., 2021).

RNA-Seq library preparation and sequencing were outsourced to a commercial service provider (Seqplexing Multiplex SL, Valencia, Spain). The differential expression profiling study was conducted on 4 tumors and 4 matched myometria obtained after surgery (T0) and long-term culture (LT-culture), when cells repopulated the tissue slices (T-15, T-20, T-25, and T-29). This sample size is consistent with current standards for exploratory transcriptomic studies aimed at identifying differentially expressed genes, particularly when using paired designs that substantially reduce inter-individual variability (Liu et al., 2014; Ching et al., 2014). The process begins with reverse transcription of 500 ng of total RNA using an oligo (dT) primer that specifically binds to the poly(A) tail of mRNA, thereby selectively capturing these transcripts. The RNA strand is then degraded, leaving a single-stranded cDNA. Next, second-strand synthesis is performed using random priming, generating a double-stranded cDNA library with incorporated unique molecular identifiers (UMIs) for further processing. Subsequently, a first PCR is performed to amplify the cDNA library, increasing the amount of genetic material available for analysis. Then, a second PCR is performed, during which an adapter is introduced to ensure that the cDNA fragments are properly anchored to the platform, enabling accurate sequence reading.

QIAxcel Advanced System assessed library quality to detect degradation or low concentration before sequencing. Sequencing was performed on the Illumina NovaSeq X with paired-end 2 × 150 bp reads. Raw FASTQ data were used for bioinformatic analysis.

### 2.10 Data analysis

The bioinformatics pipeline began with quality control and trimming of raw sequencing reads, removing adapters and poly(A) sequences. FastQC evaluated the quality of FASTQ files, ensuring good %Q20 scores and absence of errors. UMIs were processed with UMI-tools, relocating molecular markers into the read headers for accurate mapping. Reads were aligned to the “GRCh38” reference genome with STAR, accounting for splicing junctions (v2.7.10) (Dobin et al., 2013). Duplicate reads were removed using UMIs to distinguish unique molecules. Gene expression was quantified using HTSeq-count and normalized to adjust for sequencing depth. Differential expression analysis was performed with DESeq2, identifying significant gene differences. Principal component analysis (PCA) was performed in R using DESeq2; counts were normalized by the median-of-ratios method and transformed using variance-stabilizing transformation (VST). PCA was computed on the 500 most variable genes, and data visualization was performed in R using ggplot2 to generate the PC1–PC2 scatter plot with samples color-coded by group and axes annotated with the percentage of variance explained.

The expression of each gene was reported as the base-2 logarithm of the ratio of the value obtained by comparing UL vs. the matching MM at baseline (T0), the LT-culture of MM vs. the corresponding T0, and the LT-culture of UL vs. the LT-culture of the matching MM. The cutoff values for log2 fold change (log2FC) were set at 1 for upregulated and −1 for downregulated genes.

The Benjamini–Hochberg correction method for false discovery rate (FDR) was used to adjust resulting p-values for multiple testing with a cutoff of <5% (q-value).

### 2.11 Gene Ontology (GO) and KEGG pathway enrichment analysis

Gene Ontology (GO) and Kyoto Encyclopedia of Genes and Genomes (KEGG) pathway enrichment analyses were conducted to identify significantly affected biological functions and pathways associated with differentially expressed genes. Both analyses used adjusted p-values (p-adj) to assess statistical significance and calculated a Normalized Enrichment Score (NES) to quantify the direction (activation or repression) and magnitude of enrichment. GO analysis focused on categorizing changes within biological processes (BP), cellular components (CC), and molecular functions (MF), while KEGG analysis identified impacted metabolic and signaling pathways. Enriched terms and pathways with the most significant p-adj values were ranked and summarized based on the number of involved genes and their expression patterns, highlighting the most affected biological categories in the dataset.

### 2.12 Reactome enrichment analysis

Pathway enrichment analysis was performed using the ReactomePA (v1.50.0) (Yu and He, 2016) and clusterProfiler (v4.14.4) (Wu et al., 2021) packages in R. The enrichPathway function was applied to identify significantly enriched pathways from the Reactome database, using all genes from the previous differential expression analysis as input. The results were visualized using the compare Cluster function for grouped comparisons, and enrichment outcomes were displayed using the dotplot function.

## 3 Results

### 3.1 Normal and *MED12*-mutant slice cultures repopulate with smooth muscle cells

After 7 days in culture, we observed a progressive decrease in cell numbers in MM and UL slice, until they were almost completely absent. Notably, after 15–20 days in culture, the tissue slices from the four fibroids and the paired myometria were repopulated, with cells distributed across their surfaces (Supplementary Figure S1). Quantification of cellularity in normal and tumor tissues confirmed an overall decrease in cell number after day 7, followed by a subsequent increase around day 15. By day 29, cell numbers generally declined. Tumor samples exhibited a more pronounced reduction in cell number compared to normal tissues (Supplementary Figure S2).

In our previous study, we showed that immunostaining with desmin (DES) and vimentin (VIM), markers of SMCs (Wu et al., 2017), in four *MED12*-mutant UL and matched MM organ culture samples detected both markers in most cells that repopulated the culture slices. This finding confirmed that the majority of these cells were myocytes, although additional VIM+/DES- cells were also observed (Supplementary Figure S3).

*MED12* cDNA sequencing demonstrated that three tumors had a point mutation in the codon 44 hotspot (c.131 G>A, p. G44D), while one tumor presented an indel mutation in exon 2 (c.118_124del7insC, p. N40_K42insQ). All paired myometriums were wild-type (Supplementary Figures S4, S5). Sequencing chromatogram analysis indicated that the wild-type allele was essentially absent in samples L94, L98, and L100 after LT-culture. In contrast, in sample L95, the wild-type peak remained visible but was substantially lower than the mutant peak (Supplementary Figure S4). This data indicates that the mutation persisted throughout the culture period and that the transcriptomic profile was mainly consistent with that of *MED12*-mutated SMCs.

*HMGA2* expression was undetectable in all normal and tumor samples analyzed at T0. However, *HMGA2* transcripts were observed in all samples following LT-culture (Supplementary Figure S6).

### 3.2 Transcriptomic profiling reveals culture-induced transcriptional shifts while maintaining tissue-specific signatures

**FIGURE 1:**
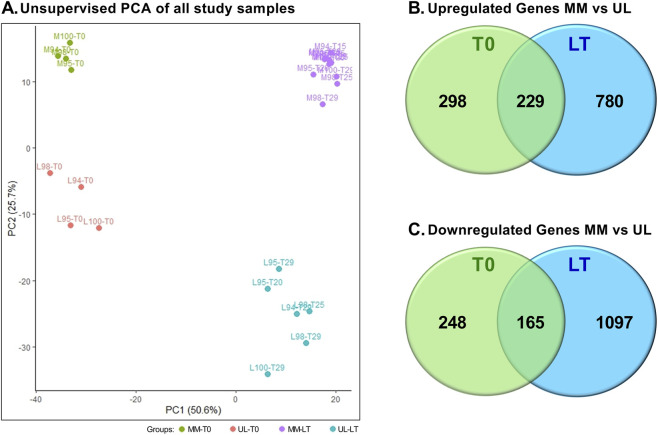
Comparative transcriptomic profiling of leiomyoma and myometrium at baseline and after long-term culture. **(A)** Principal component analysis (PCA) of all samples, including baseline (T0) and long-term cultured (LT) tissue slices, revealed a clear separation between leiomyoma (UL) and myometrium (MM) samples along the major principal components. This finding demonstrates that distinct global transcriptional profiles were preserved across culture conditions. **(B)** Venn diagram showing the overlap of upregulated differentially expressed genes (DEGs) in MM versus UL at T0 and after LT-culture. A substantial subset of genes remained consistently upregulated at both time points, indicating a conserved activation pattern throughout LT-culture. **(C)** Venn diagram illustrating the overlap of downregulated DEGs in MM versus UL at T0 and LT-culture. A shared set of downregulated genes suggests persistent transcriptional repression in leiomyoma across conditions.

To evaluate the relative contributions of tissue type and culture condition, an integrated PCA analysis was performed (Figure 1A). Principal component 1 (PC1, 50.6% of variance) primarily separated baseline (T0) tissues from LT-cultured samples, indicating that *in vitro* culture is the main driver of transcriptional variation. Principal component 2 (PC2, 25.7% of variance) distinguished myometrium from leiomyoma samples, with T0 tissues forming distinct clusters that reflect intrinsic transcriptional differences between myometrium and fibroids. After LT-culture, both tissue types remained clearly separated along PC2, demonstrating that although LT-culture induced substantial transcriptional changes, the molecular distinction between myometrium and leiomyoma was largely preserved. Notably, sample L100, which contains an indel mutation in *MED12*, exhibited a more extreme position within UL tumors, suggesting that *MED12* mutation type may influence transcriptomic profiles (Figure 1A).

To further evaluate the influence of culture conditions on transcriptional differences between fibroid and myometrium, UL and MM samples were compared at T0 and after LT-culture. Venn diagram analyses were performed separately for UL-upregulated and UL-downregulated differentially expressed genes (DEGs). At T0 and after LT-culture, 527 and 1,009 genes, respectively, were identified as upregulated in UL (Figure 1B). Among these, 229 genes remained consistently upregulated. Similarly, 413 and 1,262 genes were downregulated in UL at T0 and after LT-culture, respectively, with 165 genes persistently downregulated during LT-culture (Figure 1B). Pathways commonly overexpressed in both normal and tumor tissue at T0 and after LT-culture included ECM components and the neuronal system (Supplementary Figure S7). An overview of the 229 persistently upregulated genes revealed a broad functional profile, with significant enrichment for neuronal-related features, including synaptic components, neuronal structure, and signaling. Additionally, a substantial subset of these genes was also associated with ECM, ion channels, and genes involved in electrical signaling (Supplementary Table S1). In contrast, Reactome analysis of the downregulated genes did not identify any significantly enriched pathways at T0. Functional annotation revealed that these downregulated genes were distributed across diverse categories, including ECM-related adhesion and tissue remodeling, vascular function, immune and inflammatory signaling, and metabolic and detoxification processes (Supplementary Table S2). Collectively, these results suggest that fibroid transcriptional programs were maintained during extended *ex vivo* culture.

### 3.3 Commonly deregulated genes and pathways in fibroids were also detected in long-term leiomyoma slice cultures

**FIGURE 2:**
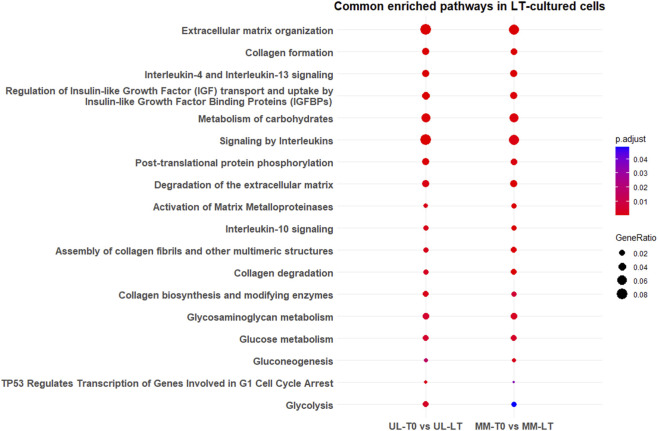
Dot plot illustrating Reactome pathways significantly enriched (q < 0.05) among genes upregulated in long-term (LT) cultured slices compared to baseline (T0) in both leiomyoma (UL) and myometrium (MM) tissue slices. Only pathways consistently significant across both tissue types are displayed. Each dot represents an enriched pathway, with size indicating the GeneRatio (proportion of input genes mapped to the pathway) and color reflecting the adjusted p-value (Benjamini–Hochberg correction). Pathways are ranked by statistical significance.

In UL LT-cultures, we found several genes and signaling pathways that have been consistently reported as dysregulated in fibroids, either compared to their respective T0 controls (no asterisk), in UL LT-cultures relative to MM LT-cultures (*), or in both comparisons (**) (Supplementary Tables S3, S4, S5). DEGs that remained consistently dysregulated (T0 and LT-culture) among normal and tumor tissue are underlined. Although the *Regulation of Insulin-like Growth Factor (IGF) transport and uptake by Insulin-like Growth Factor Binding Proteins (IGFBPs)* pathway was upregulated in both UL and MM LT-cultures (Figure 2), important gene expression differences were detected. Thus, genes uniquely upregulated in UL included *IGF1*, a known mitogen and differentiation factor involved in UL growth (Gkioka et al., 2015; Serna et al., 2018) (FC = 4, q = 1.8E-13), IGF1R* (FC = 2.5, q = 1E-18), *IGF2R* (FC = 3, q = 3E-15), IGFBP5** (FC = 4, q = 2.5E-43), *IGF2BP2* (FC = 4, q = 1.8E-03), IGFBP7-AS1* (FC = 3.5, q = 6E-04), IGFBPL1* (FC = 4, q = 3.8E-02), IGFL2* (FC = 14, q = 2.2E-13), IGFL3** (FC = 36, q = 4.1E-17), IGFL4* (FC = 15, q = 4E-03), *IGFN1* (FC = 11, q = 1.1E-08), whereas IGFBP1** (FC = 48, q = 2.3E-02), *IGFBP3 (*FC = 7.5, q = 1E-04) and IGFBP6* (FC = 2, q = 1E-03) increased expression only in MM LT-cultures. Specifically, *IGFBP5*, one of the most significantly upregulated genes in the dataset, has been found to be upregulated at both the mRNA and protein levels in UL (Cirilo et al., 2013).

The Wnt signaling pathway has been strongly implicated in leiomyoma formation (El Sabeh et al., 2021), and several members were upregulated in UL LT-cultures. Notably, WNT3* (FC = 3; q = 2.5E-02), with potential to interact with frizzled class receptors to mediate signal transduction to the nucleus, such as FZD1* and FZD7*, which were also upregulated (FC = 2; q = 8.8E-06 and FC = 3; q = 2.4E-09, respectively). In addition, we observed elevated expression of cyclin D1 (CCND1*) (FC = 2; q = 1E-03), a well-established downstream target of Wnt. In response to hypoxia, beta-catenin increased mRNA stabilization of SNAI2* (FC = 4.0, q = 2.7E-11) (D’Uva et al., 2013). Secreted frizzled-related protein 1 (SFRP1*, FC = 31; q = 1.2E-46) and the NKD inhibitor of Wnt signaling pathway 1 NKD1* (FC = 15, q = 3.5E-20) were among the most significantly upregulated genes in our UL-LT culture dataset, corroborating previous findings (Mehine et al., 2016; El Sabeh et al., 2021). Finally, *DKK2* modulator of Wnt signaling (FC = 21 q = 9.8E-08) and *MEST* (FC = 4, q = 9.4E-08), which acts as a negative regulator of Wnt/β-catenin signaling by limiting β-catenin stabilization (Song et al., 2025; Jung et al., 2011), also showed significantly increased expression in UL LT-culture. The elevated expression of upstream ligand and receptors (*WNT3*, *FZD1*, and *FZD7*), along with the canonical downstream target *CCND1*, suggests activation of the Wnt pathway in UL LT-cultures. Simultaneous upregulation of inhibitors such as SFRP1, NKD1, and DKK family members supports this interpretation, as Wnt signaling often induces its own negative feedback regulators (Clevers and Nusse, 2012).

There is strong evidence that alterations in the Retinoid Acid-signaling pathway contribute to UL development and growth, with alcohol dehydrogenase 1 (*ADH1*) and aldehyde dehydrogenase (*ALDH1)* downregulated, while the cellular retinoic acid-binding protein 2 (*CRABP2*) expression was upregulated (Tsibris et al., 2002; Arslan et al., 2005; Catherino and Malik, 2007; Zaitseva et al., 2007; Holdsworth-Carson et al., 2014). We detected ADH1B** (FC = −121, q = 6.2E-08), *ALDH1A1* ** (FC = −14, q = 5.1E-07), ALDH1A3** (FC = −7.5, q = 5E-44), *ALDH5A1* (FC = −3, q = 5E-03), *ALDH6A1* (FC = −3, q = 1.5E-08), and *ALDH7A1* (FC = −3, q = 3.3E-12) downregulated while CRABP2* (FC = 5, q = 6.64E-11) increased expression in UL LT-cultures.

UL is considered highly responsive to ovarian steroid hormones, as it exhibits increased expression of sex steroid receptors. Additionally, aromatase expression by tumor cells is thought to play a key role in the growth and maintenance of leiomyomas. In agreement with these findings, we observed upregulation of estrogen receptor 1 (ESR1*, FC = 3, q = 3.2E-33), progesterone receptor (PGR*, FC = 3, q = 1.3E-10), and aromatase (CYP19A1*, FC = 8, q = 3E-03) when comparing MM and UL LT-cultures.

An overwhelming body of evidence linked increased expression of growth factors, particularly platelet-derivate growth factors (PDGF) (Di Lieto et al., 2002; Liang et al., 2006; Wolańska and Bańkowski, 2007; Hwu et al., 2008; Mesquita et al., 2010; Koohestani et al., 2013; Islam et al., 2018), fibroblast growth factors (FGF) and their two receptors, *FGFR1* and *FGFR2* (Anania et al., 1997; Wolańska et al., 2012; Bodner-Adler et al., 2016; Jokinen et al., 2025) to enhanced cell proliferation and ECM accumulation in fibroids. In our dataset, PDGFC* was one of the five most significantly upregulated genes in UL LT-cultures (FC = 6, q = 8.5E-66), while *PDGFA* (FC = −2.5, q = 9E-04) and *PDGFD* (FC = −5, q = 1.3E-12) were repressed in MM T0 over MM LT-cultures. In addition, FGF14* (FC = 3, q = 3.1E-12) and the two receptors, FGFR1* (FC = 2, q = 2.1E-12) and FGFR2* (FC = 2.5, q = 1.5E-04), were also upregulated in UL LT-culture.

Recently, high upregulation of the tryptophan (Trp) 2,3-dioxygenase (*TDO2*) gene has been detected in *MED12-*mutated UL compared to matched myometrium, with functional studies indicating an essential role in tumor growth (Chuang et al., 2021; Hutchinson et al., 2022; Zuberi et al., 2023). Consistent with these reports, we found a strong upregulation of *TDO2* mRNA (TDO2*, FC = 15, q = 2.5E-05) in UL LT-cultures.

Overall, these findings suggest that LT-cultures of fibroids faithfully recapitulate the characteristics of the original tumors, providing a robust and unique experimental platform for studying the mechanisms underlying tumor proliferation and growth.

### 3.4 Stem cell markers were differentially expressed in the myometrium and the leiomyoma long-term culture

Hypoxia is a widely accepted trigger of SC activation (Ono et al., 2007; Mohyeldin et al., 2010). Transcriptomic profiling revealed that, in LT-culture, hypoxia-related pathways were activated in both UL and MM tissues compared to their respective T0 (Supplementary Figures S8, S9). In UL samples, GO enrichment analysis identified a significant upregulation of pathways related to hypoxia*,* with the *response to oxygen levels* among the top 5 pathways with the most significant adjusted p-values (q = 8.3E-06). Additionally, KEGG analysis revealed overrepresentation of the *HIF-1 signaling pathway* (q = 1.1E-03). Similarly, KEGG analysis (Supplementary Figure S8) revealed significant enrichment of *HIF-1 signaling* in the LT-culture of myometrium (q = 1.6E-03). Under hypoxic conditions, hypoxia inducible factor 1 (HIF1), composed of the α (HIF1A) and β (ARNT) subunits, acts as a central transcription factor regulating cellular adaptation to oxygen deprivation. HIF1 promotes the transcriptional upregulation of *CA9* and *CA12*, two carbonic anhydrases that contribute to pH regulation in the hypoxic microenvironment (Chiche et al., 2009). Analysis of the dataset demonstrated that *CA9* and *CA12* were strongly and significantly upregulated in both UL (*CA9*, FC = 286, q = 3.9E-12; *CA12*, FC = 2, q = 1E-05) and MM LT-cultures (*CA9*, FC = 237, q = 1.9E-11; *CA12*, FC = 8, q = 3.3E-20) (Supplementary Tables S3, S4, S5). *SNAI2*, a HIF1-induced transcription factor (D’Uva et al., 2013), was also significantly upregulated in UL LT-cultures (FC = 4, q = 2.7E-11, Supplementary Table S5). The long non-coding RNAs (lncRNAs) *HIF1A-AS1* (FC = 14, q = 2E-03) and *HIF1A-AS3* (FC = 28, q = 7.3E-38) were strongly upregulated in MM LT-cultures, while *HIF1A-AS3* (FC = 27, q = 3.6E-22) was also markedly induced in UL LT-cultures (Supplementary Tables S3, S4). Both lncRNAs are hypoxia-responsive genes and, depending on the cellular context, may enhance *HIF1A* expression (Reichelt-Wurm et al., 2025). In addition, in MM LT-cultures, *HIF1A* was significantly upregulated (FC = 3.5, q = 3.6E-14), whereas *ARNT2*, which can also form heterodimers with *HIF1A*, was upregulated in UL LT-cultures (FC = 2, q = 1.6E-04) (Supplementary Tables S4, S5). These findings suggest that restricted oxygen diffusion during long-term culture may create a hypoxia-like microenvironment within the tissue slices, potentially activating SSCs.

Substantial evidence supports the involvement of HMGA genes, including *HMGA1* and *HMGA2*, in stem cell proliferation and maintenance of stemness (Mas et al., 2015b; Sumter et al., 2016; Mansoori et al., 2021; Paul et al., 2024). We detected both genes highly upregulated in normal and tumor cells after LT-cultures. Notably, *HMGA1* showed the highest statistical significance among upregulated genes in the UL LT-culture dataset (Table 1). *HMGA2* is an upstream regulator of the zinc-finger transcription factor *PLAG1*, which is involved in cell growth and differentiation (Klemke et al., 2014; Gasperoni et al., 2024). Accordingly, we observed significant upregulation of *PLAG1* in LT-culture of UL (FC = 5, q = 1.9E-05) and normal myometrium (FC = 4, q = 2,2 E−04) (Supplementary Tables S3, S4). Our previous study detected strong immunostaining for HMGA2 in UL LT-cultures and fewer, weaker signals in MM LT-cultures (Supplementary Figure S10).

**TABLE 1:** Differential Upregulation of Progenitor- and Stemness-Associated Genes in Long-Term Slice Cultures.

Upregulated genes	UL		MM	
	FC	q-value	FC	q-value
HMGA1	12.5	1.4E-43	13	6.3E-27
HMGA2	16.5	3.2E-16	43	5.9E-11
ITGA2	6	9.8E-08	5	3.2E-11
**ITGA2-AS1**	​	​	10	2E-02
**ITGA3**	5	1.8E-12	​	​
**ITGA11**	2	2.6E-05	​	​
**ITGAL**	​	​	2.5*	9.6E-02
ITGAX	5	1.4E-04	9.5	1.7E-10
**ITGB1-DT**	​	​	3	1.6E-02
**ITGB2**	​	​	3**	2.9E-05
**ITGB2-AS1**	​	​	7*	4.5E-02
**ITGB5**	3	2E-27	​	​
ITGB7	7	4.1E-02	9**	1.3E-04
KLF3-AS1	2	1.5E-03	3	6.7E-06
**KLF5**	5**	5.3E-05	​	​
**HOXA13**	28*	4E-16	​	​
**HOXA11-AS**	2*	2.1E-06	​	​
**HOXA-AS2**	​	​	4	2.1E-02
**HOXB2**	​	​	2*	5.6E-03
**HOXB3**	​	​	4*	2.1E-09
**HOXB5**	​	​	9*	2.9E-10
**HOXB6**	​	​	7**	1.2E-07
**HOXB7**	​	​	10*	8.4E-06
**HOXB8**	​	​	27.5**	2.5E-06
**HOXB9**	​	​	4*	6.8E-02
**HOXC4**	​	​	2.5*	4.6E-02
**HOXC8**	​	​	6.5*	4.1E-03
**HOXC9**	​	​	4**	2.3E-03
**HOXD8**	​	​	2*	1.1E-02
**HOTAIR**	​	​	10*	6E-03
**SOX4**	​	​	6	2.4E-32
**SOX6**	​	​	2*	5.7E-03
**SOX7**	​	​	9**	1.5E-08
SOX9	8	7.6E-03	17.5	1E-02
**SOX10**	​	​	9*	2.3E-02
SOX11	3	5.1E-02	58	1.1E-06
**SOX17**	​	​	4*	2.5E-03
**SOX18**	​	​	7**	6.2E-07
**CD24**	9*	4.5E-05	​	​
**CD34**	​	​	5.5*	1.6E-03
**CD73 (NT5E)**	2*	9.8E-06	​	​
**CD90 (THY)**	2.5*	8.1E-05	​	​
**KIT**	​	​	7**	6.8E-13
**KITLG**	​	​	2.5*	5.6E-04

Genes were overexpressed in UL and MM LT-cultures compared to their respective T0 controls (no asterisk), in UL LT-cultures relative to MM LT-cultures (*), or in both comparisons (**). In the latter case, fold-change (FC) and adjusted p-value (q-value) correspond to the most statistically significant comparison. Genes exclusively upregulated in UL or MM are highlighted in bold.

Integrin genes (*ITG*) play roles in stem cell proliferation, adhesion, and lineage differentiation. CD49b, encoded by *ITGA2* and previously identified in UL SSCs, was detected in both UL and MM LT-cultures (Table 1). Interestingly, MM cells exhibited a unique and marked overexpression of the long non-coding RNA *ITGA2-AS1*. Previous immunostaining analysis detected CD49b expression in a few MM cells and in clusters of cells in UL LT cultures (Supplementary Figure S11). Three integrins were specifically upregulated in the UL LT-culture, with *ITGB5* ranking as the fifth most significantly upregulated gene in the dataset (Table 1). Conversely, integrins *ITGAL*, *ITGB2* and the lncRNAs *ITGB1-DT* and *ITGB2-AS1* were uniquely upregulated in MM LT-culture (Table 1).

*KLF5*, a pluripotency-associated Krüppel-like factor (Xiang et al., 2024) was uniquely upregulated in UL LT-culture (Table 1).

Homeobox (HOX) genes, known for their critical roles in orchestrating embryonic development and tissue differentiation, exhibited distinct expression profiles between UL and MM LT-cultures. Only *HOXA13* and *HOXA11-AS* were significantly upregulated in UL LT-culture (Table 1). In contrast, a wide array of HOX genes, including members of the *HOXB*, *HOXC*, and *HOXD* clusters, along with the lncRNA *HOTAIR*, were markedly upregulated in MM LT-culture (Table 1).

The SRY-related HMG-box (SOX) transcription factors play a critical role in maintaining stem cell identity and regulating lineage commitment (Stevanovic et al., 2021). Notably, six SOX genes were uniquely upregulated in MM long-term cultures, with four of them showing highly significant adjusted p-values (Table 1).

Classical SC or progenitor cell markers already found to be upregulated in UL were also overexpressed in our dataset, including *CD24*, *CD73*/*NT5E*, and *CD90*/*THY1*, whereas *CD34* was upregulated in MM LT-culture. Interestingly, *KIT* expression was uniquely increased in MM LT-culture slices, as was *KITLG* ligand (Table 1). Consistently, our previous study detected CD24, CD73, and Ki-67 immunostaining in UL LT-culture, whereas MM stained exclusively for KIT and showed few positive CD24 and Ki-67 cells (Supplementary Figure S11).

Collectively, these data show that long-term culture was associated with enrichment of hypoxia-related pathways and distinct stemness- and progenitor-associated gene expression profiles in leiomyoma and myometrium.

### 3.5 Differential metabolic gene activation in myometrium and leiomyoma long-term culture

Reactome enrichment pathway analysis revealed that LT-cultured cells activated central carbohydrate metabolism pathways in both UL and MM (Figure 2), however, each tissue engages a distinct gene repertoire. In UL LT-cultures, *GAPDH*, *PCK1*, *PGM1*, *HKDC1*, *SORD*, *GBE1*, and *GYS1* were upregulated, whereas MM LT-cultures preferentially upregulated *HK3*, *PC,* and *PCK2*. UL uniquely activated genes related to the degradation of complex carbohydrates (*MANBA*, *HYAL3*), while MM upregulated genes involved in sugar transport and processing (*AKR1B1*, *SLC37A2*, *SLC37A4*, *SLC35B2*). Pathways involved in transmembrane transport of bile salts, organic acids, metal ions, and amine compounds via solute carrier (SLC) proteins were specifically activated in UL LT-cultures (Supplementary Figure S12A). *PLIN2*, encoding the lipid-droplet protein Perilipin 2, was upregulated in MM LT-cultures compared to UL LT-cultures (FC = 3, q = 1.5E-10, Supplementary Table S5). Conversely, the metabolic gene ATP Citrate Lyase, *ACLY*, was upregulated in UL LT-cultures (FC = 2, q = 2.3E-08, Supplementary Table S3).

### 3.6 Long-term culture uncovers differential ECM remodeling in myometrium and leiomyoma

A defining hallmark of uterine fibroids is their abundant, disorganized ECM, which contributes to increased tissue stiffness and the characteristic fibrotic appearance of this tumor (Islam et al., 2018). Transforming growth factor beta (TGFB) plays a pivotal role in driving fibrotic processes and promoting leiomyoma proliferation (Norian et al., 2009; Ciebiera et al., 2017; Kamalipooya et al., 2021; Navarro et al., 2021; Park et al., 2022). In UL LT-cultures, we found significant upregulation of *TGFB1* (FC = 2.5, q = 5E-04), *TGFB2* (FC = 6, q = 1.2E-25), and *TGFB3* (FC = 2, q = 2.4E-06), as well as the transforming growth factor beta-induced *TGFBI* (FC = 6, q = 1.7E-12) (Supplementary Tables S3, S5). On the contrary, MM LT-cultures over T0 (Supplementary Table S4) showed downregulation of transforming growth factor beta 1 induced transcript 1, *TGFB1I1* (FC = −2.1, q = 3E-06), and the TGFB receptors *TGFBR1* (FC = −2, q = 1.1E-07), *TGFBR2* (FC = −3, q = 2.9E-36), and *TGFBR3* (FC = −6, q = 2.14E-22). Elevated *TGFB* levels have been linked to reduced expression of the collagen-binding protein dermatopontin (DPT), which is consistently downregulated in leiomyomas (Islam et al., 2018; Catherino et al., 2004; George et al., 2019) and in our dataset (FC = −4; q = 2.7E-07, Supplementary Table S3). Consistent with reports that *TGFB3* induces *CCN2* and *FN1* expression, both genes were upregulated in UL-LT cultures (*CCN2,* FC = 2.5, q = 1E-05; *FN1,* FC = 2, q = 1.3E-07, Supplementary Table S1). Activin-A, a member of the TGFB superfamily encoded by *INHBA*, was also significantly upregulated (FC = 4, q = 3.8E-06, Supplementary Tables S3, S5). *GDF6* (or *BMP13*), a member of the TGF-β superfamily known to regulate extracellular matrix production, including proteoglycan synthesis (Clarke et al., 2014; Tam et al., 2025), was also markedly upregulated (FC = 13, q = 6.6E-18).

Eight core ECM-related pathways were upregulated in both tissues when comparing T0 samples to LT-cultures (Figure 2). However, several TGFB-responsive genes were uniquely activated in UL LT-cultures, including *SERPINE1*, *PLAU*, *PLAUR*, *THBS1*, and *ADAMTS2* (Supplementary Tables S3, S5). Additional genes from fibrinolysis, TSR-domain glycosylation, and keratan sulfate synthesis pathways (such as *SERPINE2*, *FMOD*, and *ACAN*) were also upregulated, consistent with broader TGFB-driven ECM remodeling. Interestingly, the fibrosis-suppressing factors *KLF10* and *KLF11* (Spittau and Krieglstein, 2012; Lorenzo et al., 2022) showed opposite regulation patterns in long-term culture: *KLF10* was upregulated in MM (FC = 2, q = 3.4E-04,Supplementary Table S4), whereas *KLF11* was downregulated in UL (FC = −2, q = 6E-02, Supplementary Table S3).

### 3.7 Immune surveillance and angiogenesis in the myometrium are absent in the leiomyoma long-term culture

**FIGURE 3:**
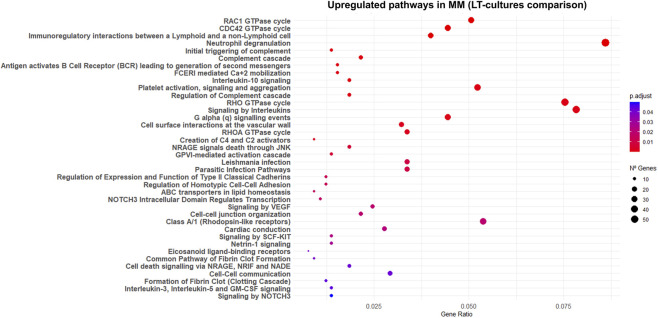
Reactome pathways upregulated in myometrium long-term cultures. The dot plot illustrates Reactome pathways that were significantly enriched (q < 0.05) among genes upregulated in cultured myometrial explants, comparing normal and tumor tissues at LT-culture (UL-LT vs. MM-LT). Each point represents a pathway, with size proportional to the GeneRatio (fraction of upregulated genes annotated to that pathway) and color indicating the adjusted p-value (Benjamini–Hochberg correction). Pathways are ordered by significance (most significant at the top).

Transcriptomic analysis comparing UL LT-cultures to MM-LT cultures revealed enrichment of immune and vascular-related pathways in MM (Figure 3). Upregulated pathways included both innate and adaptive immune processes. Interestingly, the Phospholipid Phosphatase 3 (*PLPP3*), which is involved in vascular homeostasis, was the most significantly downregulated gene in the UL dataset (FC = −6, q = 1.9E-47, Supplementary Table S3), whereas *CD274* (*PD-L1*), an immune inhibitory ligand that enables tumor cells to evade immune surveillance and promotes tumor growth (Masugi et al., 2017), was significantly upregulated in UL LT-cultures (FC = 3, q = 1E-02, Supplementary Table S5).

### 3.8 Upregulation of contractile and excitability-related programs in UL cultures

**FIGURE 4:**
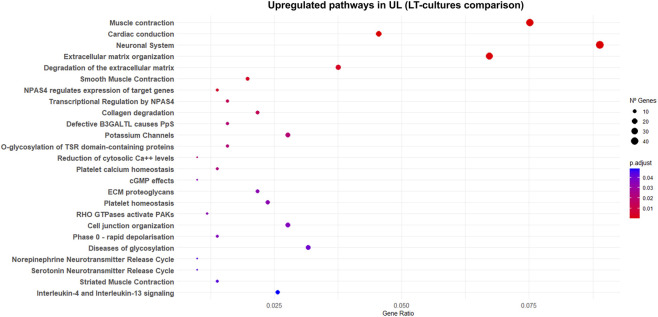
Reactome pathways upregulated in fibroid long-term cultures. The dot plot illustrates Reactome pathways that were significantly enriched (q < 0.05) among genes upregulated in cultured leiomyoma explants, comparing normal and tumor tissues at LT-culture (UL-LT vs. MM-LT). Each point represents a pathway, with size proportional to the GeneRatio (fraction of upregulated genes annotated to that pathway) and color indicating the adjusted p-value (Benjamini–Hochberg correction). Pathways are ordered by significance (most significant at the top).

In UL-LT compared with MM-LT, we observed a highly significant upregulation (q < 10^–7^) of genes associated with contraction and cytoskeletal integrity, including *TAGLN*, *CNN1*, *ACTA2*, *CACNA1C,* and *ACTG2* (Supplementary Table S5), all previously identified as myocyte-specific markers in single-cell transcriptomic analyses of UL and MM (Goad et al., 2022; Wang et al., 2023). UL-LT cultures also showed upregulation of pathways related to muscle contraction, which were not detected in the normal-tumor comparison at T0. In contrast, the *neuronal system* pathway was overexpressed at T0 and in LT-culture, with several genes implicated in electrical excitability, ion channel activity, cytosolic Ca^2+^ regulation, and depolarization processes (Figure 4; Supplementary Table S1). This pathway also included genes involved in vesicle trafficking and Ca^2+^-dependent exocytosis. Conversely, MM-LT cultures displayed enrichment of pathways involving small GTPase signaling (RAC1, CDC42, RHO, and RHOA GTPase cycles), Gα(q)-mediated signaling, ABC transporters in lipid homeostasis, Class A/1 rhodopsin-like GPCRs, and eicosanoid ligand-binding receptors (Figure 3). Overall, UL-LT cultures are enriched for smooth-muscle contractile and excitability-related signatures, whereas MM-LT cultures preferentially activate signaling pathways linked to GTPase activity and GPCR signaling.

## 4 Discussion

In this study, we exploited the distinctive capacity of LT-organ cultures of MM and UL to spontaneously induce stem cell proliferation and differentiation within a physiologically relevant context. By preserving native stem-cell niches and cell-cell and cell-ECM interactions, transcriptomic profiling of LT-cultures enabled the identification of previously unrecognized genes and pathways driving metabolic reprogramming, fibrosis, smooth muscle cell contractility, aberrant vascularization, and immune evasion in *MED12*-mutated fibroid SSCs. While LT culture induced substantial transcriptional changes in both tissue types, a significant proportion of gene expression differences distinguishing fibroid from myometrium at baseline (T0) persisted after LT culture. This persistence suggests that the transcriptional identity of fibroid tissue is maintained even after extended culture. The validity of this platform is further supported by the identification of numerous genes and pathways that are frequently deregulated in fibroids, thereby establishing LT-organ culture as a robust and faithful model of fibroid disease biology.

Multiple lines of evidence indicate that the observed pattern of cell loss followed by robust repopulation in long-term cultures aligns with the survival and activation of a small subset of *MED12*-mutant stem/progenitor cells, supporting the hypothesis that fibroids originate from a mutated SSC (Bulun, 2013; Commandeur et al., 2015; Khadka et al., 2025). First, hypoxia-related pathways, which are established activators of SSCs, particularly myometrial SSCs (Ono et al., 2007), were enriched in UL and MM LT-culture (Supplementary Figures S8, S9). Canonical hypoxia-induced genes included *CA9*, which promotes the survival and proliferation of cancer stem cells, and *SNAI2*, which suppresses lineage commitment and differentiation while promoting mammary stem/progenitor cell states (Lock et al., 2013; D’Uva et al., 2013). Both genes were upregulated in UL LT-cultures. Notably, CA9 enables tumor cells to survive in hypoxic, acidic, and nutrient-poor environments and is considered a promising anticancer therapeutic target (Ronca and Supuran, 2024). Second, in LT-cultures, *ITGA2* mRNA, encoding CD49b, was strongly upregulated compared to T0, and immunostaining detected CD49b-positive cells in UL and MM LT-culture (Supplementary Figure S11). This surface marker has previously been used to isolate SSCs from fibroids, and characterization demonstrated expression of canonical stemness genes, including *OCT4*, *KLF4*, and *NANOG*. Isolated SSCs also form colonies *in vitro* and regenerate tumors *in vivo*. Third, *HMGA1* and *HMGA2* were substantially overexpressed in LT-cultures. Both genes are well-established regulators of self-renewal, chromatin plasticity, and stem-cell identity, and have been consistently found to be upregulated in UL (Gross et al., 2003; Klemke et al., 2009; Shah et al., 2012; Parisi et al., 2020; Paul et al., 2024). Fourth, CD24 and CD73 surface markers were highly upregulated at both mRNA and protein levels, with most UL cells staining positive for both markers in UL LT-cultures (Supplementary Figure S11). CD24 is frequently detected in progenitor or tumor-initiating cells across various malignancies, and previous studies have reported strong CD24 expression in UL, with a significant myocyte population exhibiting abundant CD24 expression and co-expressing CD73, a mesenchymal stem cell marker (Drosh et al., 2014). Moreover, CD24 was persistently upregulated in both T0 and LT-culture. Fifth, the *MED12*-mutant allele, present at T0 in all four tumors, remained preferentially expressed in LT-cultures. This finding is significant, as in traditional two-dimensional primary cultures, *MED12*-mutant cells gradually decrease, even without passaging, resulting in cultures predominantly composed of tumor-associated fibroblasts (TAFs), which are abundant in *MED12*-mutated fibroids (Wu et al., 2017). TAFs lack the driver mutation and exhibit a higher capacity to attach and proliferate *in vitro*, overtaking the culture dish and displacing *MED12*-mutant SMCs (Nadine Markowski et al., 2014; Bloch et al., 2017; Wu et al., 2017). The persistence of *MED12* mutations in cultures suggests that components of the tumor microenvironment, including ECM and other tumor-associated cells, may be required to support the proliferation and differentiation of *MED12*-mutated SSCs. A previous study found *MED12* mutations in UL stem-like cells isolated using the Hoechst 33342 efflux assay. These cells promoted the *in vivo* growth of leiomyoma xenograft tumors, resulting in much larger tumors when mixed with myometrial SMCs (Ono et al., 2012). Additionally, paracrine signals sent from mature myometrial or leiomyoma cells to stem cells further enhance stem cell regeneration and proliferation, ultimately promoting *in vivo* tumor expansion (Ono et al., 2013).

An alternative hypothesis regarding the origin of fibroids proposes that uterine leiomyomas (UL) arise from differentiated myometrial smooth muscle cells that acquire driver mutations. Experimental evidence demonstrates that *HMGA2* overexpression in myometrial cells induces proliferative, dedifferentiated, and tumor-initiating phenotypes characteristic of fibroid development (Mas et al., 2015b; Paul et al., 2024). Additionally, the introduction of *MED12* Gly44 mutations in SMCs reproduces several cellular, transcriptional, and metabolic alterations observed in fibroids (Buyukcelebi et al., 2023). Notably, none of the tumors at T0 exhibited *HMGA2* expression, although all four tumors harbored *MED12* mutations and showed *HMGA2* upregulation after LT culture. Interestingly, *HMGA2* expression is transiently induced following stem-cell activation, remains elevated during the proliferative myoblast phase, and declines as cells undergo terminal differentiation during skeletal muscle development (Li et al., 2012). In LT-cultures, HMGA2 immunoreactivity was detected in the nuclei of cells within localized regions of tissue slices. This pattern suggests that, *in vivo*, slowly proliferating fibroids may not consistently exhibit detectable *HMGA2* expression, as *HMGA2* is restricted to stem/progenitor cells, which constitute approximately 6% of the fibroid cell population (Yin et al., 2015). In contrast, in long-term cultures where these cells are activated, expanded, and subsequently differentiated, the proliferative phase is captured, facilitating detection of *HMGA2* upregulation. Accordingly, elevated *HMGA2* expression has been reported in a subset of *MED12*-mutant tumors, and transcriptional analyses demonstrate substantial overlap between the *MED12*-and *HMGA2*-defined subtypes (Mehine et al., 2016; George et al., 2019).

Stem cells self-renew through symmetric division or generate two daughter cells with distinct fates *via* asymmetric division. Maintaining a proper balance between these division models is essential for tissue homeostasis, while disruption may contribute to pathological outcomes such as tumorigenesis (Majumdar et al., 2020). In the mouse myometrium, c-Kit-positive cells are located near SSCs and function as a transient amplifying population that progresses toward terminal differentiation (Szotek et al., 2007). Immunostaining of MM LT-cultured slices revealed very few cells immunoreactive for CD49b, the KIT receptor, a stem cell marker (Rossi et al., 2000), HMGA2, and the proliferation marker Ki67 (Supplementary Figure S11). These findings suggest that, in human myometrium, stem cell proliferation is tightly controlled and that, similar to the mouse, KIT-expressing cells may correspond to a transit-amplifying cell population. Restricted proliferation in MM may be mediated, at least in part, by regulation of the surface integrin CD49b, as *ITGA2-AS1*, which negatively regulates *ITGA2* (CD49b) expression (Verhoeff et al., 2022), was selectively upregulated in MM LT-cultures (Table 1). Conversely, the presence of clusters of CD49b-immunopositive cells, abundant expression of the progenitor cell markers CD24 and CD73, and widespread Ki67 expression suggest dysregulated proliferation of SSC/progenitor cells in fibroids. Notably, CD24 remained persistently upregulated in fibroids at both T0 and after LT-culture, a surface marker that promotes tumor growth, invasion, and metastasis in various cancers (Duex et al., 2017). Collectively, these findings support the notion that LT-culture reveals a tightly controlled stem cell–mediated proliferative program in normal myometrium, whereas *MED12*-mutated SSCs in fibroids exhibit dysregulated proliferation.

Comparison of MM and tumor tissue at T0 did not show enrichment of pathways associated with muscle contraction in UL (Supplementary Figure S7). In contrast, upregulation of these pathways was detected in UL LT-culture (Figure 4). These differences may reflect a loss of contractility in MM during LT-culture, rather than an intrinsic increase in UL. This observation aligns with the MM adopting a phenotype similar to early pregnancy, which is characterized by increased proliferative activity (hyperplasia), reduced differentiation, and decreased contractility (Shynlova et al., 2009). On the other hand, the persistent enrichment of the *neuronal system* pathway in fibroids compared with MM (Supplementary Figure S7) suggest increased electrophysiological responsiveness and calcium-coupled signaling capacity, as well as a greater potential for regulated vesicle-mediated secretion in fibroids.

Stem-cell proliferation and differentiation are energetically demanding processes, and tissue slices depleted of cells by day 7 become almost fully repopulated by day 25, indicating robust energy-dependent expansion. Metabolic reprogramming, however, differed markedly between normal and tumor tissue: fibroid cells displayed a metabolic profile centered on glycogen turnover, complex-carbohydrate degradation, and broad SLC-mediated transport, whereas myometrial cells relied on pathways that facilitate glucose uptake, entry into glycolysis, and the conversion of simple sugars into metabolic intermediates. Notably, the concomitant downregulation of *PLIN2* and upregulation of *ACLY* in UL is metabolically consistent with a reprogrammed energetic state. Reduced *PLIN2* expression destabilizes lipid droplets and limits lipid storage capacity, thereby enhancing fatty-acid mobilization, mitochondrial oxidation, and glycolytic activity in fibroids (Okeigwe et al., 2019). This shift increases cellular demand for cytosolic acetyl-CoA, a requirement that is met by upregulating *ACLY*, the principal enzyme that converts citrate to acetyl-CoA to sustain *de novo* lipogenesis, membrane biosynthesis, and histone acetylation in proliferating cells. Thus, the low *PLIN2* and high *ACLY* signature reflects a coordinated metabolic adaptation that supports growth and survival in leiomyoma cells. Divergent metabolic programs reveal potential metabolism-based therapeutic approaches to restrain fibroid cell proliferation, including the inhibition of nutrient-uptake transporters such as SLC proteins, which have shown therapeutic potential in cancer (Nwosu et al., 2023; Zhang et al., 2026). *ACLY* inhibition induces proliferative arrest across multiple cancer models (Zaidi et al., 2012; Bader et al., 2025a), and several pharmacological *ACLY* inhibitors are currently being evaluated in preclinical studies, with some of them already advancing into early-phase clinical trials (Bader et al., 2025b).

The abundant ECM is a hallmark of UL, and several ECM-related pathways remained consistently upregulated in T0 and LT cultures (Supplementary Figure S7). TGFB is highly involved in the pathogenesis of UL (Ciebiera et al., 2017). Several genes directly regulated by TGFB involved in fibrosis were upregulated in UL LT-cultures, whereas TGFB receptors were downregulated in MM LT-cultures. Notably, three TGFB-responsive transcription factors involved in fibrosis were deregulated in UL. *KLF10* acts as a negative feedback regulator of TGFB signaling, suppressing TGFB-mediated stellate cell activation and fibrogenesis in the liver, whereas *KLF10* deficiency enhances fibrosis and impairs skeletal-muscle function (DiMario, 2018; Lee et al., 2020; Hwang et al., 2023). *KLF10* also represses *ATF3,* a pro-fibrotic transcription factor that promotes ECM accumulation and myofibroblast activation (Hwang et al., 2023). In MM LT-cultures, *KLF10* was upregulated, concomitant with a marked downregulation of *ATF3* (FC = −8, q = 1.2E-08, Supplementary Table S2). Similarly, *KLF11* functions as a negative regulator of fibrotic pathways (Spittau and Krieglstein, 2012; Lorenzo et al., 2022) and was downregulated in UL LT-cultures, consistent with its reported epigenetic silencing in UL (Fernandez-Zapico et al., 2003; Navarro et al., 2012). In contrast, *KLF5* was upregulated in LT-cultures (Table 1) and, under hypoxic conditions, interacts with hypoxia-inducible factor 1α to promote lung cancer proliferation, as well as ECM reorganization and accumulation (Luo and Chen, 2021). Moreover, *KLF5* can activate *TGFB1* transcription, thereby perpetuating TGFB signaling and reinforcing its profibrotic action (Nagai et al., 2003; Methatham et al., 2022). The combined loss of *KLF10*–mediated negative feedback and the gain of *KLF5* expression may contribute to the persistent fibrosis characteristic of UL. These observations highlight the therapeutic potential of restoring *KLF10* activity and inhibiting *KLF5* to counteract excessive ECM deposition and limit fibroid growth.

Although most cells in LT-culture are SMCs, other cell types, such as fibroblasts or myofibroblasts, may persist and contribute to ECM remodeling (Islam et al., 2018; Lan et al., 2025). Several IGF members upregulated in UL LT-culture are involved in fibroblast activation. IGF1 and IGF1R are central mediators of fibroblast proliferation, survival, and ECM protein synthesis, while IGF2R promotes fibrosis by stimulating fibroblast activation, ECM protein deposition, and myofibroblast transformation, while disrupting protease-inhibitor balance to favor matrix accumulation (Hung et al., 2013; Zhu et al., 2025). Moreover, IGF2R contributes to fibrosis by activating latent TGFB (Zhu et al., 2022). Among the IGF binding proteins, IGFBP5 has a well-established role in promoting collagen deposition, matrix stiffening, and fibroblast migration across various fibrotic conditions (Pilewski et al., 2005; Nguyen et al., 2021; Shi et al., 2025). In contrast, MM LT-cultures showed selective upregulation of *IGFBP3* and *IGFBP6*. Although IGFBP3 stimulates collagen and fibronectin synthesis, thereby contributing to ECM accumulation (Pilewski et al., 2005), IGFBP6 has been implicated in limiting fibroblast proliferation and ECM deposition, serving as a counter-regulatory mechanism (Liso et al., 2022). Overall, dysregulation of IGF genes in UL points to excessive ECM accumulation and sustained fibrotic activity, whereas MM expression profile suggests a more balanced regulation of ECM, with both pro-fibrotic (*IGFBP3*) and potentially protective factors (*IGFBP6*) shaping tissue homeostasis.

Interactions between cells and the ECM regulate HOX gene expression, thereby modulating cell proliferation, adhesion, apoptosis, and migration. Reciprocally, ECM components are also controlled by HOX genes, a process referred to as dynamic reciprocity (Leppert, 2012). Notably, several genes from clusters B and C, including the lncRNAs *HOTAIR* and *HOXA-AS2*, were uniquely upregulated in MM LT-culture (Table 1). Conversely, *HOXA11-AS* and *HOXA13* were upregulated in UL LT-cultures. *HOXA13* plays a critical role in ECM regulation during limb development (Williams et al., 2005), and its overexpression in UL has been previously reported (George et al., 2019; Leistico et al., 2021). In UL, *HOXA13* was shown to be hyperacetylated at H3K27, a modification associated with increased expression of several ECM-related genes (Carbajo-García et al., 2022). Silencing of *HOXA11-AS* has been reported to upregulate HOXA11 (Guo et al., 2019), whereas *Hoxa11* knockdown in the mouse genital tract altered both mRNA and protein levels of type I and type III interstitial collagens (Ma et al., 2012). Taken together, the persistent upregulation of *HOXA11-AS* and *HOXA13* observed in UL at T0 and LT-culture may contribute to aberrant synthesis and expression of ECM-related genes, including *COL10A1*, *COL12A1*, and *COL23A1*, which were also persistently upregulated in UL T0 and LT-cultures (Supplementary Tables S1, S3, S5). These findings underscore the role of HOX gene regulation in driving ECM remodeling in normal MM and UL, and suggest that the ECM-HOX axis is a promising therapeutic target for disrupting fibrotic progression in UL.

Integrin receptors constitute the primary molecular interface between cells and the ECM and also contribute to cell-cell interactions. The gene pairs *ITGA2-ITGB1*, *ITGA3-ITGB1*, *ITGAV-ITGB5*, and *ITGA11-ITGB1* encode the integrin heterodimers α2β1, α3β1, αVβ5, and α11β1, respectively, with one member upregulated in UL (*ITGA2*, *ITGA3*, *ITGB5*, and *ITGA11,* Table 1). These receptors bind key ECM components, including laminin, fibronectin, and collagen, thereby regulating cell-matrix adhesion, collagen organization, and ECM remodeling (Heino, 2016; Morandi et al., 2016; Zeltz et al., 2022). In contrast, *ITGB1BP2* (melusin), a β1 integrin-binding protein with essential roles in cytoskeletal organization, muscle fiber maturation, and mechanotransduction in muscle cells, was downregulated in MM LT cultures (FC = −10, q = 3.6E-28). In addition, the αLβ2 integrin receptor (encoded by the *ITGAL-ITGB2* gene pair) was upregulated in MM, accompanied by increased expression of the antisense lncRNA *ITGB2-AS1*, which has been reported to enhance *ITGB2* expression (Liu et al., 2018; Zu et al., 2022). αLβ2 is a receptor for multiple intercellular adhesion molecules (ICAMs) and is predominantly expressed on immune cells, where it facilitates immune cell adhesion and trafficking, promotes T-cell activation, enhances natural killer and cytotoxic T-cell-mediated cytotoxicity, and contributes to macrophage-mediated clearance of apoptotic cells (Crozat et al., 2011; Kristóf et al., 2013; Walling and Kim, 2018). Consistent with this, markers associated with monocytes/macrophages (*CD14, CD163, CD163L1, CD68, CD36, CD300A, CD300LB,* and *CD33*), lymphocytes (T, B, and NK cells), and dendritic cells (*CD4, CD22, CD28, CD38, CD40, CD48, CD52, CD74,* and *CD84*) were selectively upregulated in MM LT-cultures (Supplementary Table S5), suggesting a higher presence of immune cells in myometrial cultures. Collectively, these findings suggest that the integrin expression profile in UL enhances cellular anchorage, promotes collagen remodeling, and favors tissue stiffness and immune evasion, whereas MM appears to support an ECM environment permissive to immune cell proliferation and activity. In line with this evidence, recent hypotheses propose that fibroid growth is associated with the acquisition of an immunosuppressive phenotype that facilitates excessive ECM deposition (Saad et al., 2024). Targeting integrin signaling or disrupting ECM-integrin interactions may therefore represent promising therapeutic strategies to limit cellular expansion and fibroid growth.

Canonical endothelial cell markers, such as *PECAM1* (CD31), *VWF*, and *CDH5* (CD144), were upregulated in MM LT-cultures (Supplementary Table S5). Consistently, MM LT-cultures displayed transcriptional programs indicative of vascularization, with multiple members of the SOX family (*SOX4*, *SOX6*, *SOX7*, *SOX10*, *SOX17,* and *SOX18*) significantly upregulated (Table 1). These transcription factors share structural and functional similarities and act cooperatively to regulate vascular development and maintain endothelial homeostasis (Zhou et al., 2015; Wang et al., 2017; Vervoort et al., 2018; Ling et al., 2025). Conversely, *PLPP3,* a key regulator of vascular homeostasis through its control of endothelial cell adhesion, migration, and inflammatory signaling (Busnelli et al., 2018), was downregulated in UL LT-cultures. Taken together, these findings support a model in which myometrial growth is coupled to coordinated tissue vascularization, whereas UL appears to evade or impair blood vessel formation, consistent with the defective and dysregulated angiogenesis characteristic of fibroids (Tal and Segars, 2014; Kirschen et al., 2021).

**FIGURE 5:**
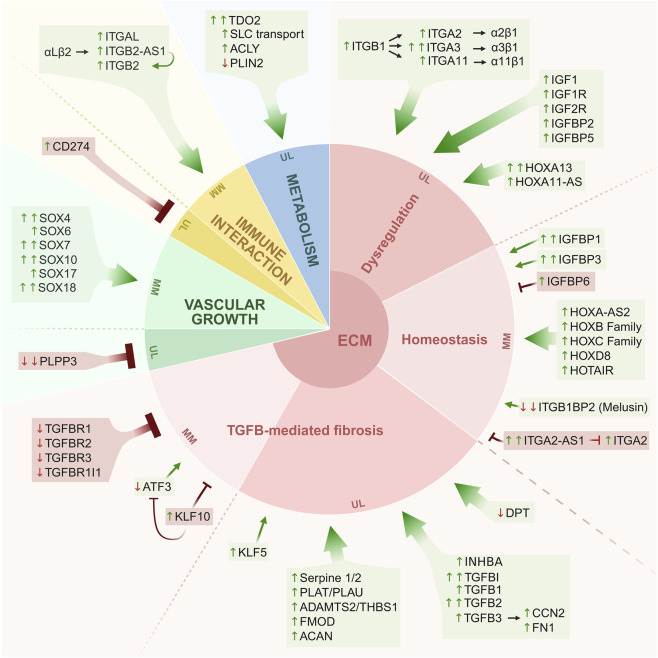
Overview of consistently dysregulated genes identified in this study, grouped by functional categories. Schematic summary of genes consistently dysregulated in UL compared with matched MM in long-term culture, organized into major functional categories. The central circle highlights extracellular matrix remodeling as a core feature, surrounded by modules related to metabolism, immune interaction, and vascular growth. Arrows preceding gene names indicate the direction of dysregulation (up- or downregulation), while T-bars represent inhibitory interactions or diminished signaling activity. Arrows linking integrin subunits denote heterodimer formation. Overall, these data highlight a coordinated transcriptional network driving uterine leiomyoma pathophysiology. Created in BioRender. Vázquez, P. (2026) https://BioRender.com/xomqrfa.

Figure 5 schematically presents the primary and novel findings of this study, offering an integrative overview of key molecular alterations. Consistently dysregulated genes are grouped into functional categories, such as metabolism, immune interactions, vascular growth, and ECM remodeling.

Some study limitations should be considered when interpreting these findings. First, the analysis is based exclusively on transcriptomic profiling, which does not directly capture protein abundance, post-translational regulation, or functional activity. Although pathway enrichment analyses consistently identified hypoxia-related signaling as a prominent feature of LT-cultures in both MM and UL, hypoxia was not directly measured, and a causal role in stem cell activation cannot therefore be formally established. Nevertheless, the robust expression of stem cell-associated transcripts observed here, together with the detection of stem cell markers by immunofluorescence, strongly supports the notion that stem cells are activated in both tissues, independent of whether hypoxia is the primary initiating stimulus.

A second limitation concerns the study’s temporal resolution. All end-point samples from long-term cultures were pooled and analyzed as a single “long-term” group, precluding a detailed temporal dissection of stem cell activation, proliferation, and differentiation. Future studies analyzing matched intermediate time points independently (e.g., T15, T20, T25, or T29 myometrium compared with their respective T0 controls and corresponding leiomyoma time points) would enable reconstruction of the differentiation process and identification of genes activated early *versus* late along the stem cell-to-myocyte axis.

Partial RNA degradation may have affected transcript coverage and should be considered when interpreting the results. Although 3′mRNA-Seq can be used with partially degraded RNA, it does not fully compensate for reduced RNA integrity. The relatively low RIN values in some samples are an inherent limitation of the study.

Finally, this study focused exclusively on *MED12*-mutated leiomyomas, the most prevalent genetic subtype of uterine fibroids. Whether the transcriptional programs and pathway dynamics described here are shared by, or distinct from, leiomyomas harboring alternative driver alterations remains to be determined and warrants investigation in future work.

Beyond leiomyomas, long-term cultures of normal myometrium offer a unique opportunity to investigate proliferation, differentiation, and ECM interactions in MM, a tissue fundamental to uterine function and reproductive health. Additionally, resident stem cells in conditions such as liver, pulmonary, or cardiac fibrosis, where slice cultures are already available, may also activate during long-term cultures, offering new insight into the mechanisms promoting fibrotic remodeling. Ultimately, this approach may reveal mechanisms with translational potential in regenerative medicine and oncology.

## Data Availability

The data discussed in this publication have been deposited in NCBI’s Gene Expression Omnibus (Edgar et al., 2002) and are accessible through GEO Series accession number GSE309174 (https://www.ncbi.nlm.nih.gov/geo/query/acc.cgi?acc=GSE309174). All data generated or analysed during this study are included in this published article and its Supplementary Material files.
