# Supplementary figures and images for: Stem cell activation in organ culture reveals novel transcriptional programs underlying metabolic, fibrotic, vascular, and immune dysregulation in uterine leiomyomas
Source: Front Cell Dev Biol. 2026 Apr 22;14:1804196. doi: 10.3389/fcell.2026.1804196 (PMC13143910; doi:10.3389/fcell.2026.1804196)

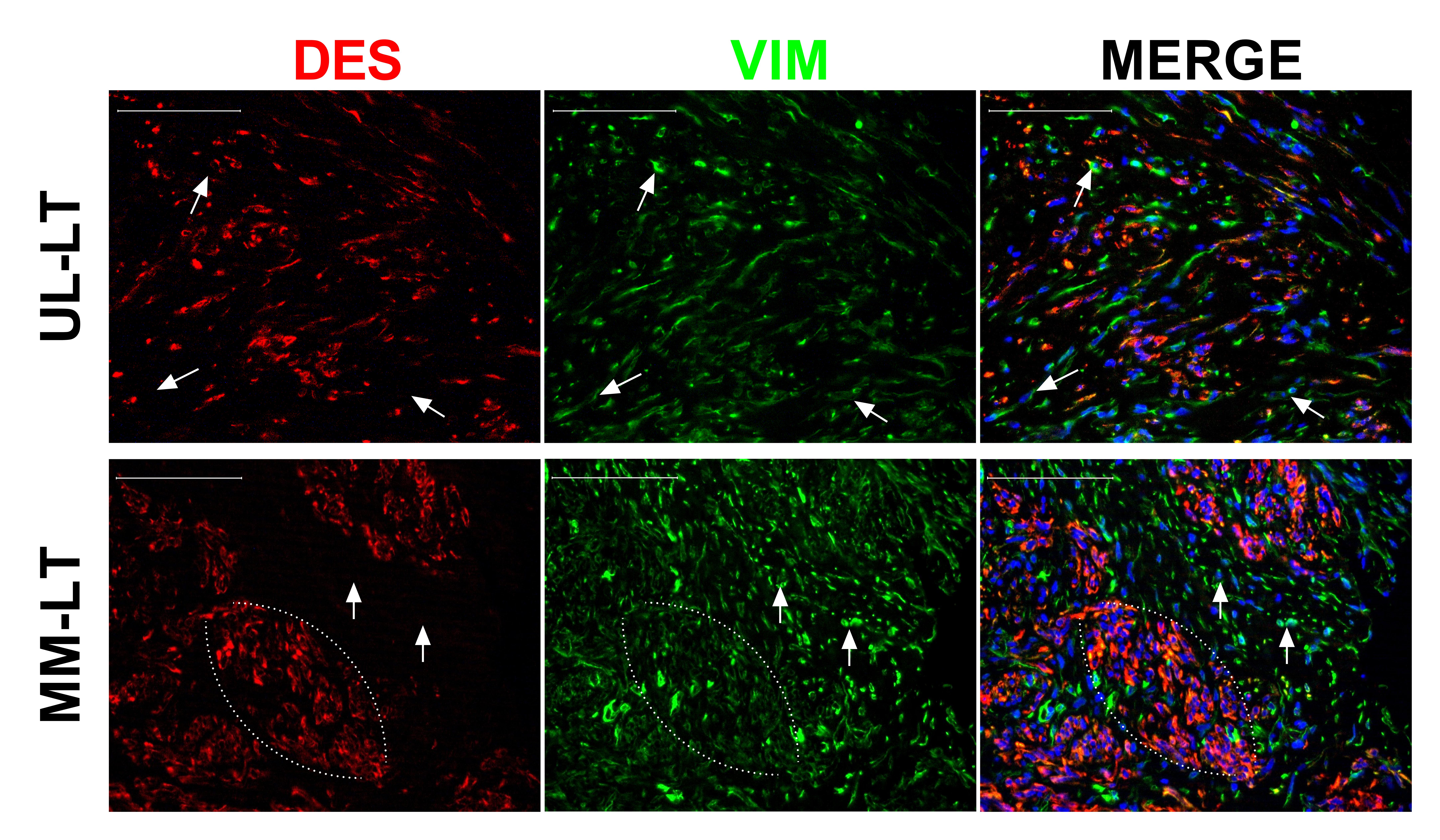

Supplement: Supplementary file 2 [file Image3.jpeg]

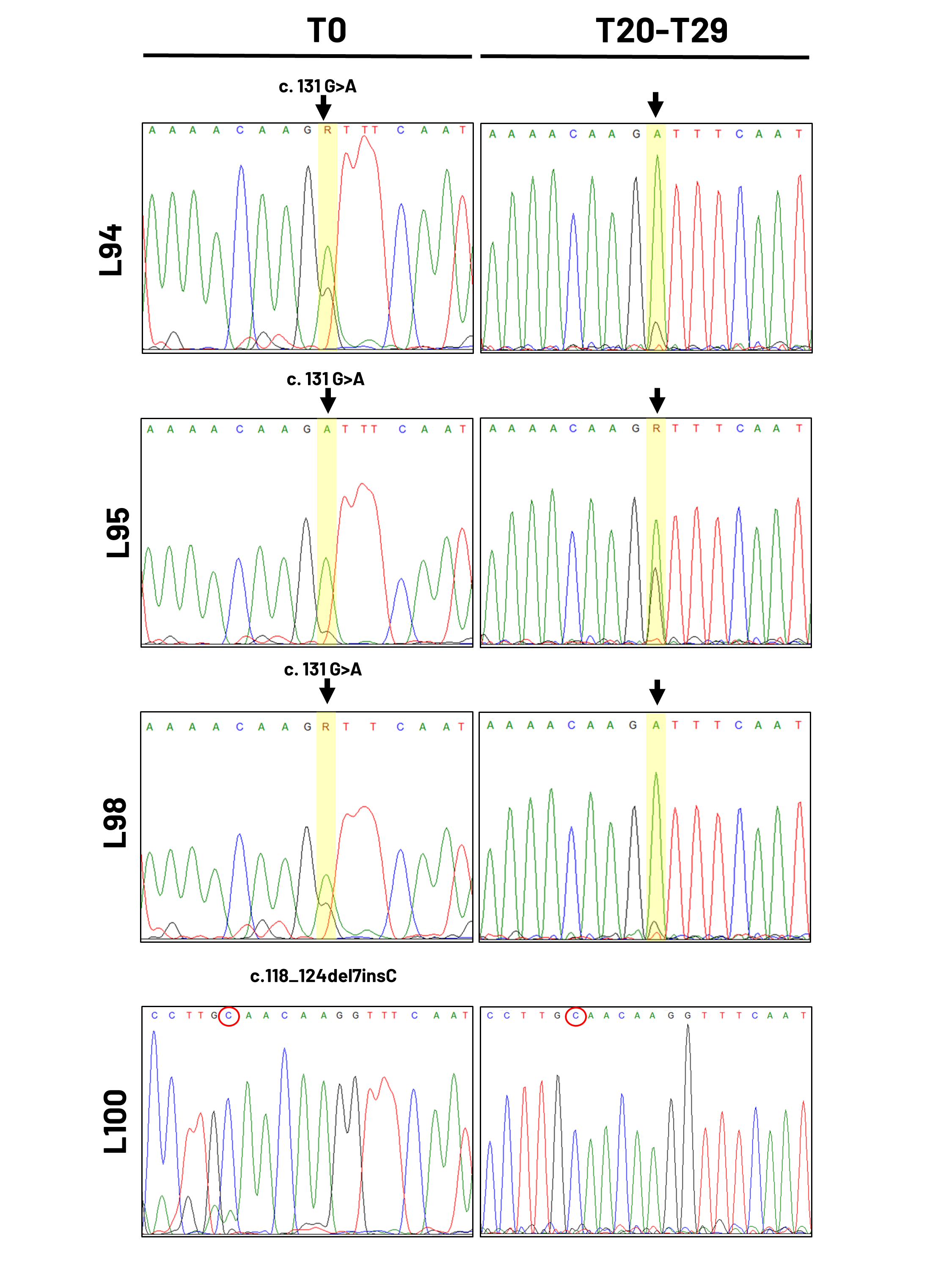

Supplement: Supplementary file 5 [file Image4.tif]

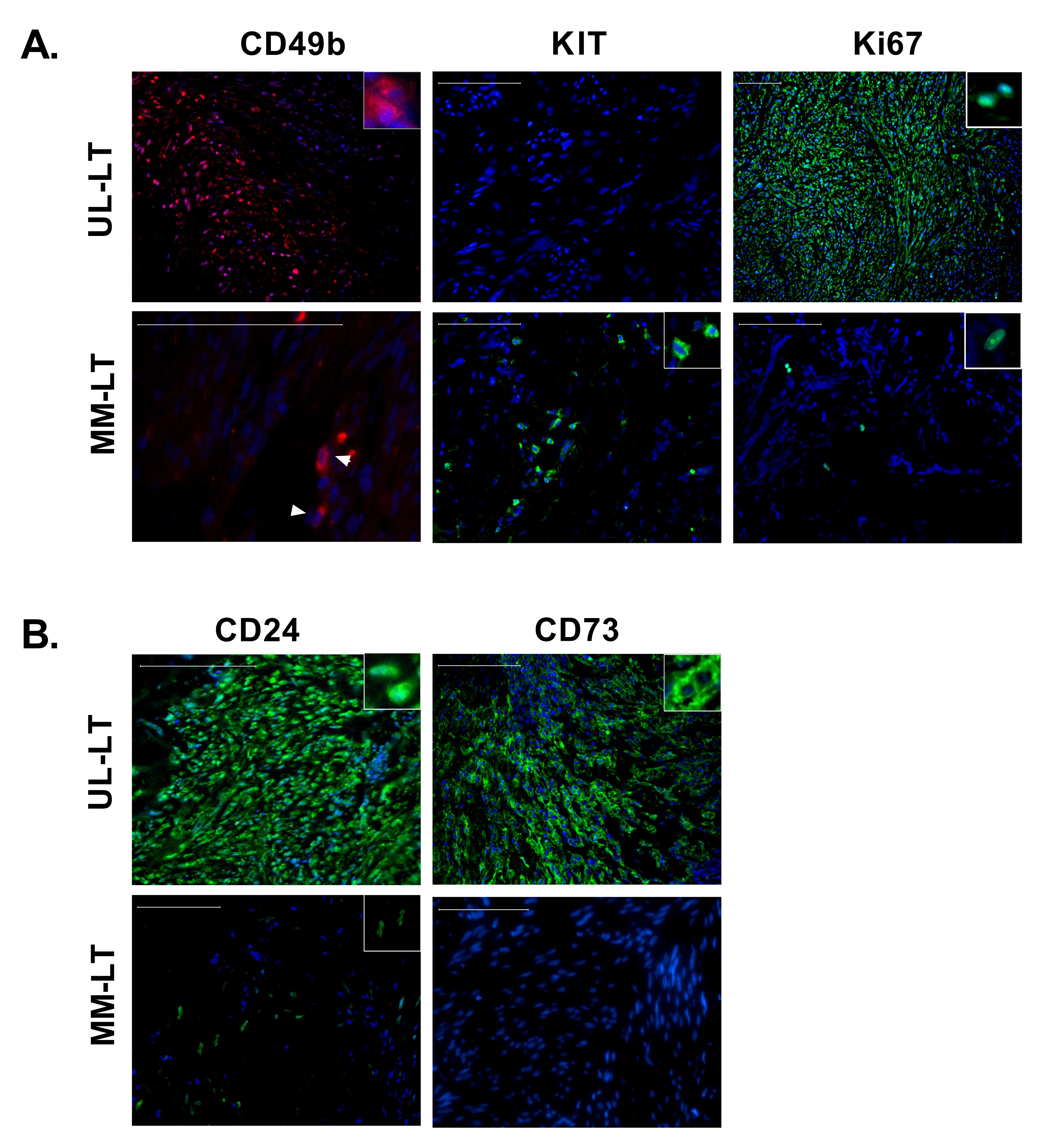

Supplement: Supplementary file 9 [file Image11.tif]

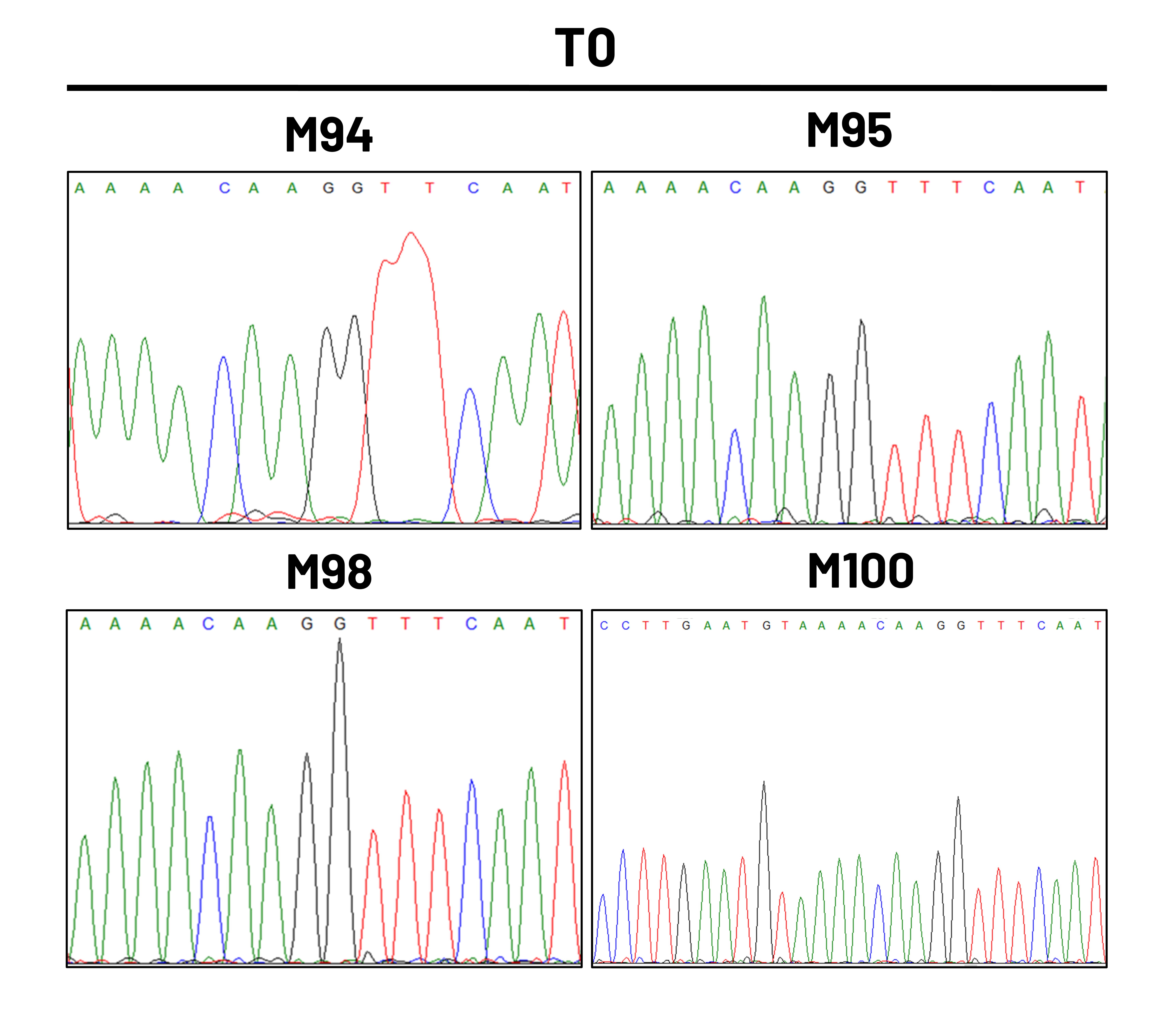

Supplement: Supplementary file 10 [file Image5.jpeg]

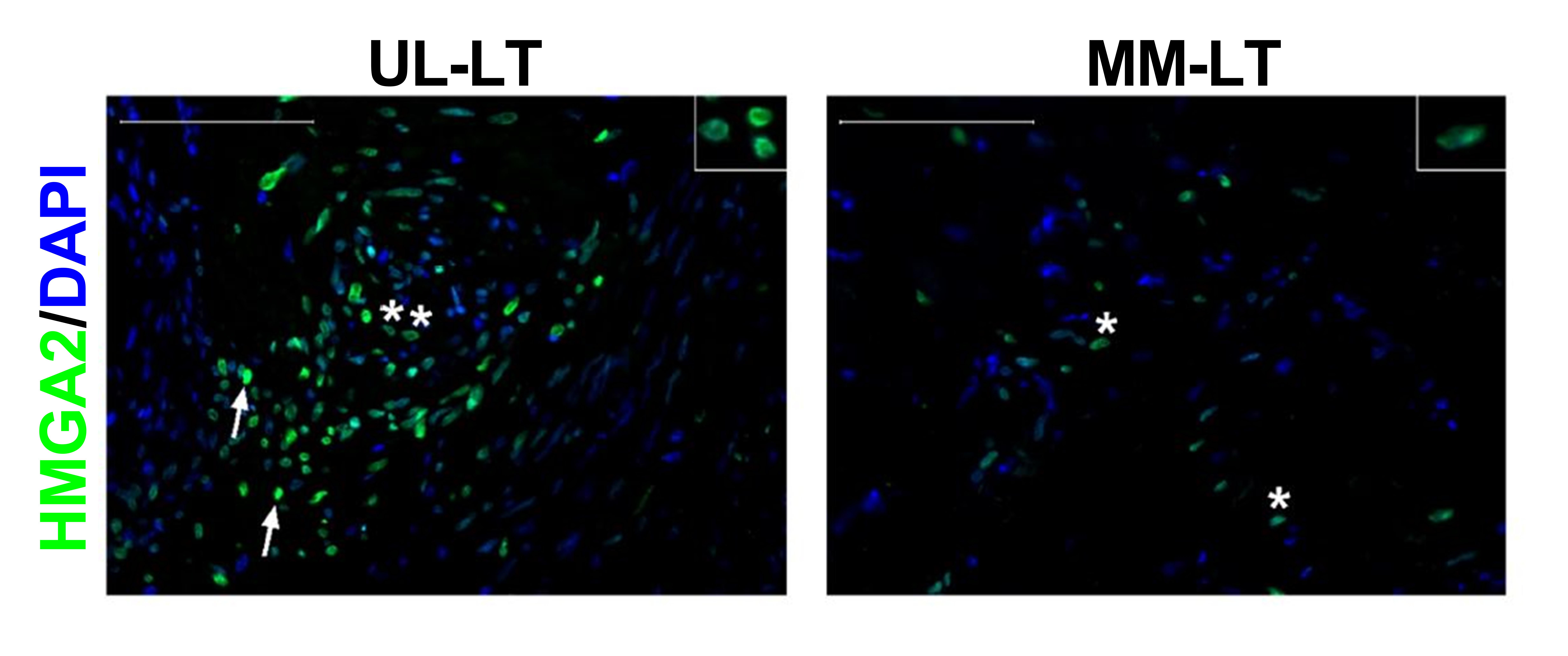

Supplement: Supplementary file 11 [file Image10.jpeg]

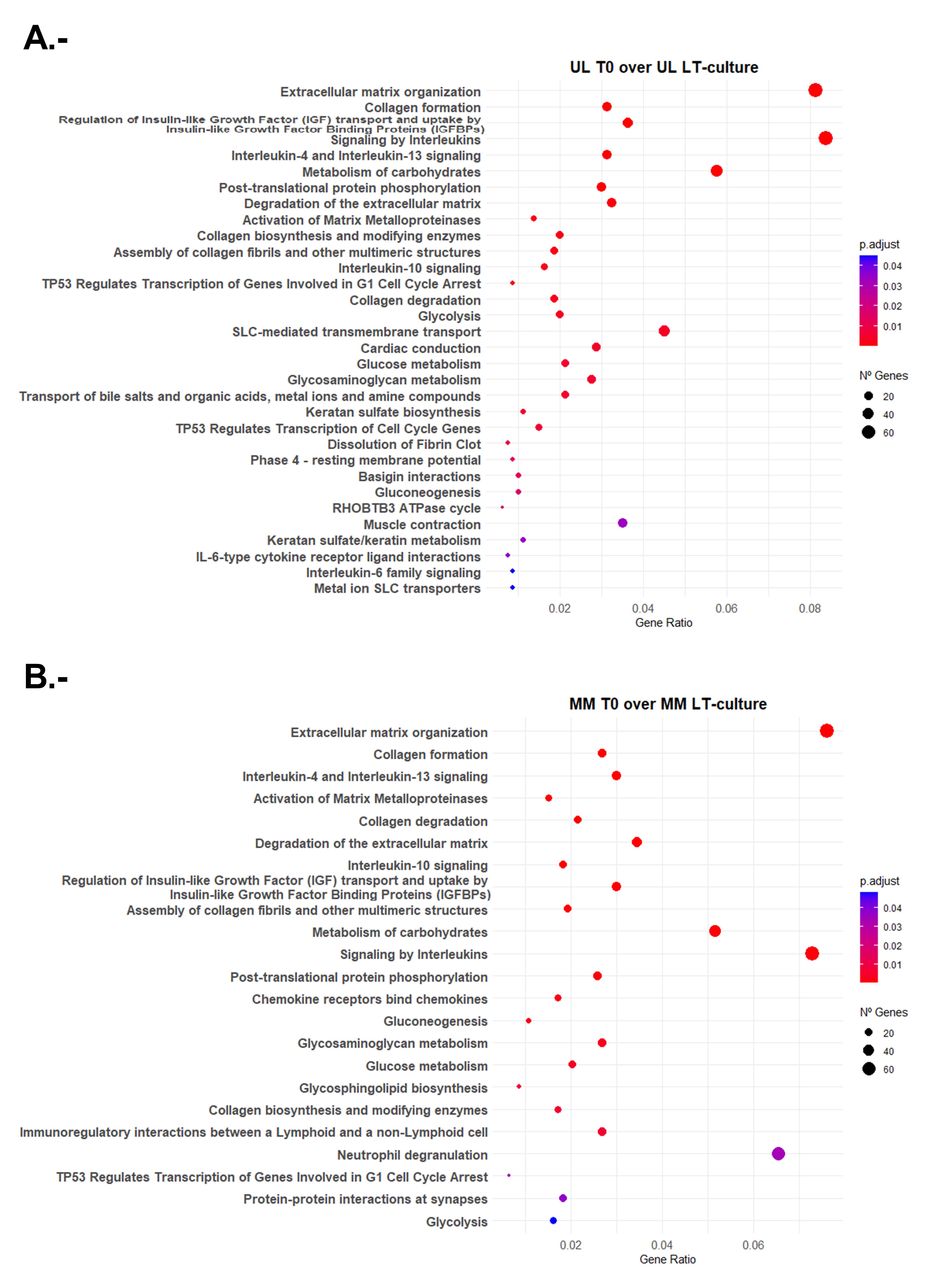

Supplement: Supplementary file 16 [file Image12.tif]

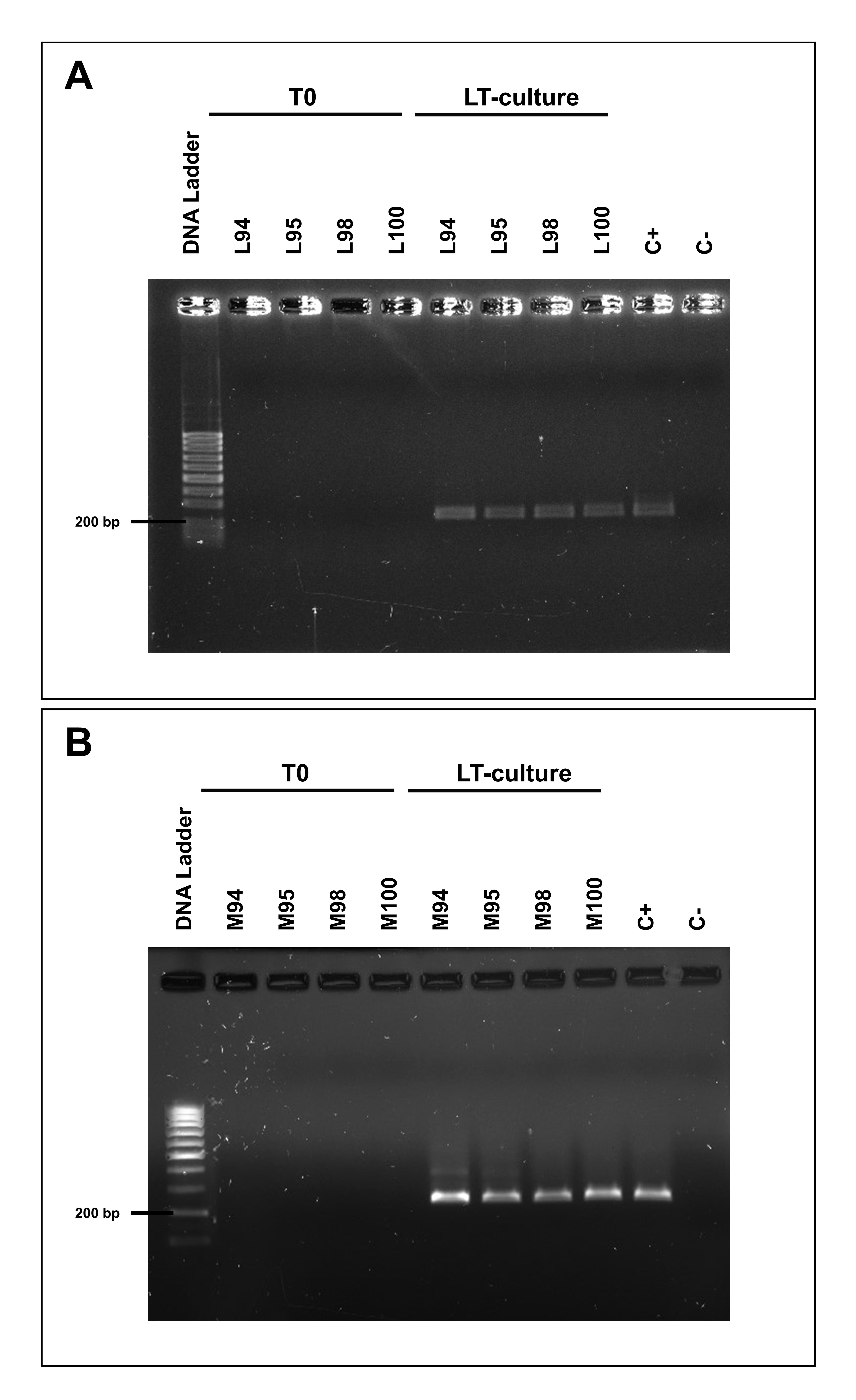

Supplement: Supplementary file 18 [file Image6.jpeg]
