# Supplementary material for: Stem cell activation in organ culture reveals novel transcriptional programs underlying metabolic, fibrotic, vascular, and immune dysregulation in uterine leiomyomas
Source: Front Cell Dev Biol. 2026 Apr 22;14:1804196. doi: 10.3389/fcell.2026.1804196 (PMC13143910; doi:10.3389/fcell.2026.1804196)
Supplement: Supplementary file 17 [file DataSheet1.docx]

***Supplementary Material***

# Supplementary Data

**Supplementary Table S1.** Excel spreadsheet listing genes upregulated in fibroids compared to myometrium at T0 and during long-term culture (LT). LT‑specific genes are those upregulated exclusively after long‑term culture, while T0‑specific genes are upregulated only at baseline (T0). Shared‑DEG refers to genes upregulated at both T0 and after long‑term culture.

**Supplementary Table S2.** Excel spreadsheet listing genes downregulated in fibroids compared to myometrium at T0 and during long-term culture (LT). LT‑specific genes are those dowregulated exclusively after long‑term culture, while T0‑specific genes are downregulated only at baseline (T0). Shared‑DEG refers to genes downregulated at both T0 and after long‑term culture.

**Supplementary Table S3.** Excel spreadsheet containing upregulated and downregulated genes in UL T0 compared with UL LT-culture

**Supplementary Table S4.** Excel spreadsheet containing upregulated and downregulated genes in MM T0 over MM LT-culture

**Supplementary Table S5.** Excel spreadsheet containing upregulated and downregulated genes in UL LT-culture over MM LT-culture

# Supplementary Figures

**Supplementary Figure S1.** Histological analysis of T0 and long-term culture slices. Representative images of leiomyoma and myometrium tissue sections, hematoxylin and eosin stained (H&E) at baseline (day 0) and after 7, 15, 20, 25, and 29 days of culture. Scale bar 100 μm.

**Supplementary Figure S2.** Quantification of cell numbers in leiomyoma (UL) and paired myometrium (MM) tissues during extended culture. Cellularity in leiomyoma **(A)** and myometrium **(B)** was quantified at baseline (day 0) and after 7, 15, 20, 25, and 29 days in culture for four paired samples (L/M94: blue circle; L/M95: red square; L/M98: orange triangle; L/M100: green rhombus). Quantification was performed in 3–4 randomly selected areas, each covering 0.0949 mm², from hematoxylin and eosin (H&E)-stained tissue sections. Data are presented as mean ± standard deviation (SD) of the number of counted cells per tissue type at each time point. Although initial cell numbers and sample variability differed, both tumor and normal tissues demonstrated a consistent temporal pattern: a decrease in cell number after 7–15 days, an increase after 15–20 days, and a subsequent decline by day 29.

**Supplementary Figure S3.** Tissue distribution of smooth muscle cells (SMCs) at long-term culture (LT) of leiomyoma (UL) and myometrium (MM) slices. DES (red) stains SMCs, whereas VIM (green) stains both SMCs and other cell types. DAPI stains the cell nucleus (blue). White arrows indicate VIM+ cells, more abundant in MM LT-culture. Scale bar 100 μm. This figure is reproduced from Salas et al., *Biomedicines*, 2022, 10(7), 1542, under CC BY license.

**Supplementary Figure S4.** Preservation of *MED12* mutation in leiomyoma (UL) across the long-term culture. Sequence electropherograms illustrating mutations in exon 2 of *MED12* in the original tumor (T0) and after long-term culture (T20, T25, and T29). Three tumors (L94, L95, and L98) showed the same point mutation, c.131 G>A, in the codon 44 hotspot (highlighted in yellow). The indel mutation of the remaining tumor (L100) consisted of the deletion of seven nucleotides (positions 118-124) and insertion of a cytosine (red circle).

**Supplementary Figure S5.** *MED12* mutation is absent in paired myometrium (MM) samples. Sequence electropherograms illustrating the sequence of exon 2 of *MED12* in myometrial samples at T0. Samples M94, M95, and M98 exhibit the wild-type sequence at codon 44, whereas M100 demonstrates the wild-type sequence across the indel mutation region.

**Supplementary Figure S6.** *HMGA2* mRNA expression in leiomyoma (UL) and myometrium (MM) at baseline (T0) and following long-term culture (LT-culture). *HMGA2* PCR products from (A) leiomyoma (L94, L95, L98, L100) and (B) myometrium (M94, M95, M98, M100) slice samples were analyzed at baseline (T0) and after long-term culture (T20, T25, T29). *HMGA2* expression was undetectable at T0 in both tumor and normal samples, while an *HMGA2* amplification band was present after long-term culture in both tissues. The first well contains a 100 bp DNA ladder. The expected size of the *HMGA2* PCR product is 196 bp.

**Supplementary Figure S7.** Dot plot illustrating Reactome pathways enriched among genes upregulated in UL compared to MM tissue slices at baseline (T0) and after long-term culture (LT-culture). Only pathways consistently upregulated at both time points are shown. For LT-culture, significance was defined as q < 0.05. For T0, the potassium channel pathway is included despite a slightly higher adjusted p-value (q = 0.08), as numerous genes related to potasssium channel were significantly upregulated at T0 (Table 1). Dot size indicates the GeneRatio (proportion of input genes mapped to each pathway), and dot color indicates the adjusted p-value (Benjamini–Hochberg correction). Pathways are ordered by statistical significance.

**Supplementary Figure S8.** Significantly overexpressed pathways in uterine leiomyoma (UL) following long-term (LT) culture compared to baseline (T0). Hypoxia-related pathways are highlighted in yellow. Pathway enrichment analysis was performed using Gene Ontology (GO) and Kyoto Encyclopedia of Genes and Genomes (KEGG) databases, with significance determined by adjusted p-values (Benjamini–Hochberg correction). The left panel displays the Normalized Enrichment Score (NES) for each pathway, indicating both the direction (upregulation) and magnitude of pathway activity changes. The right panel presents a scatter plot summarizing adjusted p-values, total gene counts per pathway, and the proportion of differentially expressed genes.

**Supplementary Figure S9.** Significantly overexpressed pathways in myometrium (MM) following long-term (LT) culture compared to baseline (T0). Hypoxia-related pathways are highlighted in yellow. Pathway enrichment analysis was performed using Gene Ontology (GO) and Kyoto Encyclopedia of Genes and Genomes (KEGG) databases, with significance determined by adjusted p-values (Benjamini–Hochberg correction). The left panel displays the Normalized Enrichment Score (NES) for each pathway, indicating both the direction (upregulation) and magnitude of pathway activity changes. The right panel presents a scatter plot summarizing adjusted p-values, total gene counts per pathway, and the proportion of differentially expressed genes.

**Supplementary Figure S10.** Immunofluorescence staining of HMGA2 (green) in UL and MM slices at LT-culture is shown. UL-LT slices display cells with strong (white arrows) and weak (asterisks) signals, whereas MM-LT slices show fewer immunostained cells with reduced HMGA2 expression (asterisks). Nuclei are counterstained with DAPI (blue). Inserts provide detailed views of HMGA2 expression in cell nuclei. Scale bar 100 μm. Adapted from Salas et al., *Biomedicines*, 2022, 10(7), 1542, under CC BY license.

**Supplementary Figure S11.** Differential expression of stem cell/progenitor cell markers in leiomyoma (UL) and myometrial (MM) tissue slices after long-term culture (LT). **(A)** Immunofluorescence staining of CD49b (ITGA2) (red), KIT (green), and the proliferation marker Ki67 (green) in UL and MM slices at LT-cultures. Nuclei are counterstained with DAPI (blue). Arrowheads highlight CD49b⁺ cells in the myometrium. **(B)** Immunofluorescence staining of CD24 (green) and CD73 (green) in UL and MM slices at LT-culture. Nuclei are counterstained with DAPI (blue). Inserts provide detailed views of marker expression. Scale bar 100 μm. Adapted from Salas et al., *Biomedicines*, 2022, 10(7), 1542, under CC BY license.

**Supplementary Figure S12.** Reactome pathways upregulated in long-term cultured uterine leiomyoma (UL) and myometrium (MM) slices compared to T0.

Dot plot illustrating Reactome pathways significantly enriched (q < 0.05) among genes upregulated in long-term cultured uterine leiomyoma (UL) slices **(A)** and myometrium (MM) slices **(B)** compared to their respective T0 (baseline). Each point represents a pathway, with size proportional to the GeneRatio (the fraction of upregulated genes annotated to that pathway) and color indicating the adjusted p-value (Benjamini–Hochberg correction). Pathways are ranked by significance, with the most significant at the top.
